# Resting-state EEG activity predicts frontoparietal network reconfiguration and improved attentional performance

**DOI:** 10.1038/s41598-020-61866-7

**Published:** 2020-03-19

**Authors:** Jacek Rogala, Ewa Kublik, Rafał Krauz, Andrzej Wróbel

**Affiliations:** 1 Bioimaging Research Center, World Hearing Center, Institute of Physiology and Pathology of Hearing, Mokra 17 street, Kajetany, 05-830 Nadarzyn Poland; 2 Instytut Biologii Doświadczalnej im. Marcelego Nenckiego, 3 Pasteur Street, 02-093 Warsaw, Poland; 3 Military University of Technology, Physical Education, 3 gen, Sylwestra Kaliskiego street, 00-908 Warsaw, Poland; 4 Department of Epistemology, Institute of Philosophy, University of Warsaw, 3 Krakowskie Przedmiescie street, 00-927 Warszawa, Poland

**Keywords:** Neuroscience, Cognitive neuroscience, Attention

## Abstract

Mounting evidence indicates that resting-state EEG activity is related to various cognitive functions. To trace physiological underpinnings of this relationship, we investigated EEG and behavioral performance of 36 healthy adults recorded at rest and during visual attention tasks: visual search and gun shooting. All measures were repeated two months later to determine stability of the results. Correlation analyses revealed that within the range of 2–45 Hz, at rest, beta-2 band power correlated with the strength of frontoparietal connectivity and behavioral performance in both sessions. Participants with lower global beta-2 resting-state power (gB2rest) showed weaker frontoparietal connectivity and greater capacity for its modifications, as indicated by changes in phase correlations of the EEG signals. At the same time shorter reaction times and improved shooting accuracy were found, in both test and retest, in participants with low gB2rest compared to higher gB2rest values. We posit that weak frontoparietal connectivity permits flexible network reconfigurations required for improved performance in everyday tasks.

## Introduction

One of the main goals of neuroscience is to understand how neuronal activity organizes behavior. To elucidate this relationship, many studies have focused on the putative links between task-related neuronal electrical activity and behavioral performance^1–5^. In parallel, BOLD (blood-oxygen-level-dependent) imaging investigations have revealed that (i) resting-state (spontaneous) connectivity correlates with functionally activated networks^6–14^, and (ii) specifically with the performance of various cognitive functions, such as attention^15^, working memory^16,17^ and fluid intelligence^18^. It has been further suggested that such correlations could be explained by the individual characteristics of brain networks^19,20^. However, the uncertain physiological origins of hemodynamic signals^21^ provide limited insight into the mechanisms governing the relationship between resting-state and task-related activations and therefore behavioral outcomes.

Recording electrophysiological activity is one way that the relationship between resting-state and behavioral performance can be directly assessed. Indeed, several investigations have already revealed correlations between specific EEG bands and different aspects of cognitive performance. For example, in the attentional domain, alpha oscillations have been proposed to clear sensory information from distractors^22^, the beta to gamma band ratio can assure critical-state dynamics for optimal information processing^23^ and alpha and beta band activity can reduce attentional investment during rest^24^. Yet, none of the proposed mechanisms have characterized the intrinsic properties of resting-state networks such as power of EEG bands and connectivity, and how they could relate to functional connectivity underlying behavioral performance.

We hypothesized that specific electrophysiological signatures of spontaneous, individual EEG activity in large-scale networks would predict cognitive performance. To address this issue, we focused on spatial attention, one of the most broadly investigated and well-understood types of cognitive performance. We used two tasks involving attention: a laboratory-based visual search and a ‘field’ gun-shooting task, both of which were repeated after two months. We analyzed the power of canonical oscillatory bands (theta, alpha, beta-1, beta-2) and the strength of the connectivity between all pairs of EEG electrodes at rest and during task performance. The only reproducible predictor of attentional performance that was valid for both tasks during test and retest session was the resting-state beta-2 band (22–29 Hz) activity averaged over all electrodes, which negatively correlated with behavioral performance. While recent studies also investigated relations between EEG activity and behavioral performance^25,26^, they did not show and discuss the modulating role of frontoparietal connectivity as it was revealed in our work.

Taking together our findings demonstrate that the average resting-state beta-2 power is an electrophysiological signature of the strength of long-range frontoparietal and frontooccipital connections. Finally, we suggest that weaker ongoing beta-2 oscillations in long-range networks facilitate plastic changes in response to cognitive load, which subsequently leads to improved behavioral performance.

## Results

### Performance in visual search and shooting tasks

To investigate the relationship between resting-state EEG and behavioral performance in both laboratory-based and ‘field’ (ecological) tests, we used two attention-related tasks: visual search and target shooting with the pneumatic gun. Both tasks were repeated within a two-month test–retest interval. Participants’ performance in the visual search task was evaluated based on reaction times and accuracy (expressed as % of correct responses), and their performance in target shooting was evaluated based on shooting scores.

Neither of the two behavioral measures of the visual search task differed between the test and retest (average reaction time: test = 1.11 ± 0.15 s, retest = 1.05 ± 0.16 s, p > 0.1; accuracy: test = 81.6 ± 9.8, retest = 83.3 ± 9.1, p > 0.3; two-tailed paired t-tests; n = 33). In the gun-shooting task, participants performed significantly better in the retest (scoring on average 285.74 ± 30.50 points out of 400) than in the test session (m = 252.28 ± 31.93 points, p < 0.01, two-tailed paired, t-test, n = 33). This improved performance probably resulted from the shooting training, which could increase their proficiency; however, we had no control group to support this hypothesis. For details on the behavioral results, see Supplementary Information S1.

### Beta-2 – the only EEG frequency band related to behavioral performance

**Figure 1:**
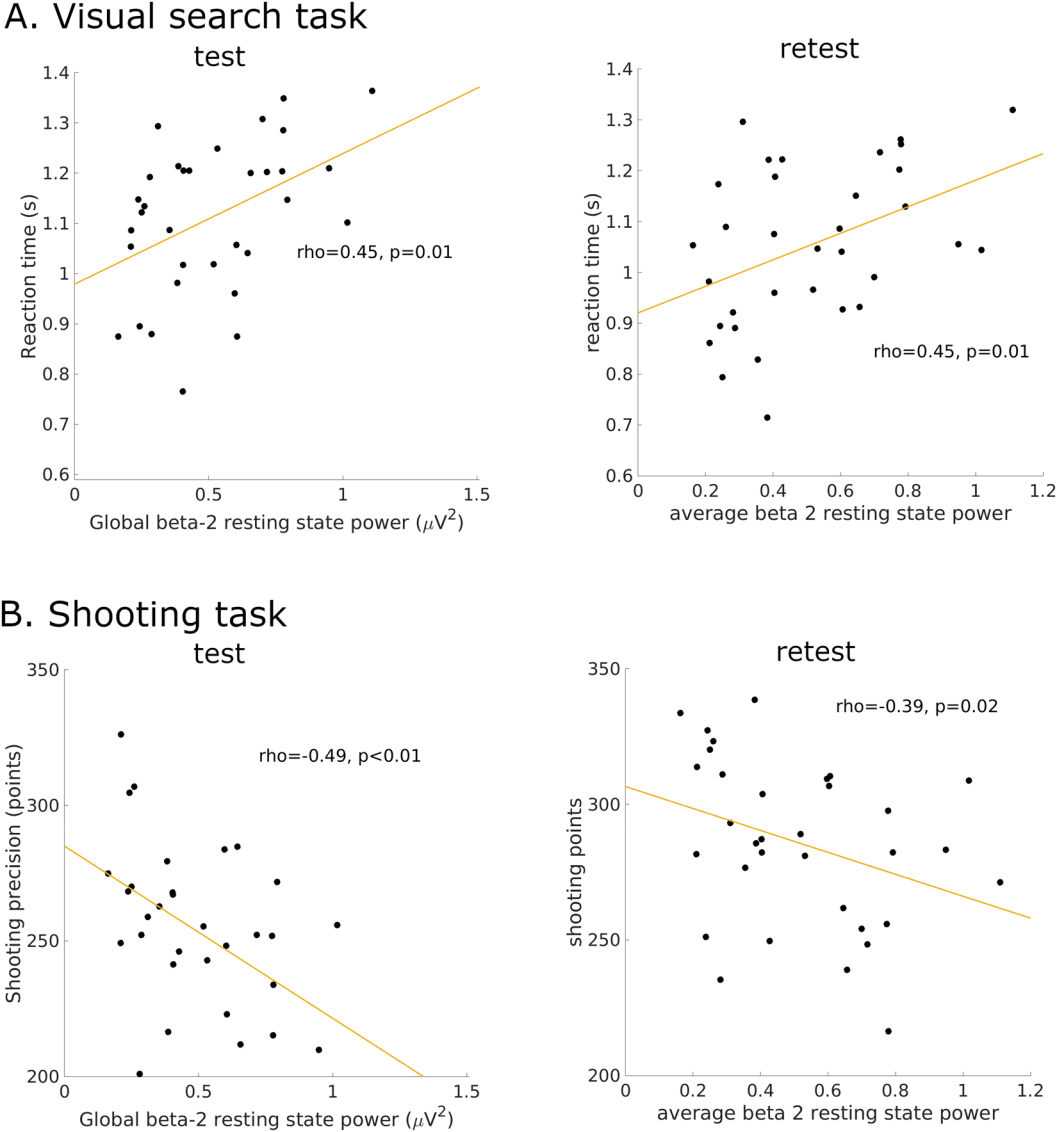
Correlations between average global beta-2 power (22–29 Hz) in the resting state (gB2rest) and behavioral performance. (**A**) Reaction times in the visual search task, n = 33. (**B**) Scores from target shooting (out of a possible 400), n = 33.

To find relationships between resting-state EEG activity and task performance, we correlated global resting-state power (for every 3 Hz window in the 2–45 Hz range over all electrodes) with individual results of accuracy and reaction times in the visual search task and with shooting scores. Two of the measures – reaction times in the visual search task and scores in the shooting task correlated significantly (p = 0.009, n = 33 and p = 0.003, n = 33 respectively for visual search and shooting task) with the same cluster of frequencies in the beta-2 range (22–29 Hz) (Fig. 1). The average power of the resting state in the beta-2 range (termed in the remaining text gB2rest) correlated positively with reaction time (Fig. 1A) and negatively with shooting score (Fig. 1B) in both the test and retest sessions. Resting-state power in other EEG ranges, defined by the moving window, did not correlate with reaction time nor with shooting scores. We did not find any correlation between gB2rest and accuracy in the visual search task; therefore, this measure was excluded from subsequent analyses.

### gB2rest versus connectivity in the resting state

Since the beta band is believed to transmit information or modulatory, attention-related signals between brain structures/cortical areas^27–29^, in our search of neurophysiological underpinnings of the observed correlations we expected to find correlations between the power of the beta band (gB2rest) and resting-state connectivity (estimated by PLVs).

**Figure 2:**
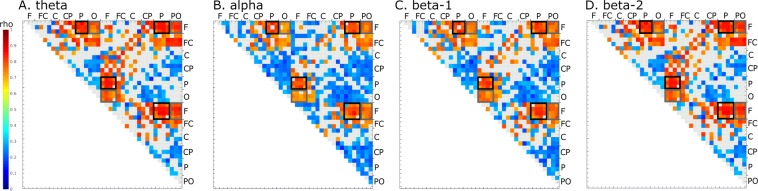
Significant correlations between gB2rest and phase-locking values (PLVs) of the resting-state measured before test session in: (**A**) theta, (**B**) alpha, (**C**) beta-1 and (**D**) beta-2 EEG bands. PLVs were calculated for averaged one second epoch. Shades of red and blue denote significant FDR-corrected correlation strength (color bar to the left) between the two signals recorded from electrodes indicated next to the box outlines. Light gray squares denote lack of significance. Gray outlines denote PLVs between frontal and occipital electrode sites; black outlines denote PLVs between frontal and parietal sites.

To analyze correlations between the power of the beta band (gB2rest) and resting-state connectivity, we averaged PLVs calculated from the test resting-state data over all participants, individually for each pair of signals (calculated for 39 electrodes in the visual search task and 19 in the shooting task) and correlated them with gB2rest. Significant correlations (p < 0.05, FDR corrected) between gB2 rest and PLVs were found for the majority of the signal pairs (out of all 741 pairs of electrodes, significant correlations in theta band were found for 495, in alpha: 487, in beta-1: 461 and in beta-2 for 410 pairs). The highest positive correlations were found for long-range frontoparietal and frontooccipital connections in the beta-2 band (with correlation values ranging from 0.48 to 0.72 for frontoparietal and from 0.38 to 0.62 for frontooccipital connections; Fig. 2 and Suppl. Inf. S2).

### gB2rest versus task-related connectivity

Functional connectivity accompanying cognitive behavior was previously found to be highly correlated with the resting-state networks in fMRI studies^30–33^, therefore having established the relationship between gB2rest and the pattern of resting-state intrinsic connectivity, we used the same approach to determine putative relationship between gB2rest and task-related connectivity.

**Figure 3:**
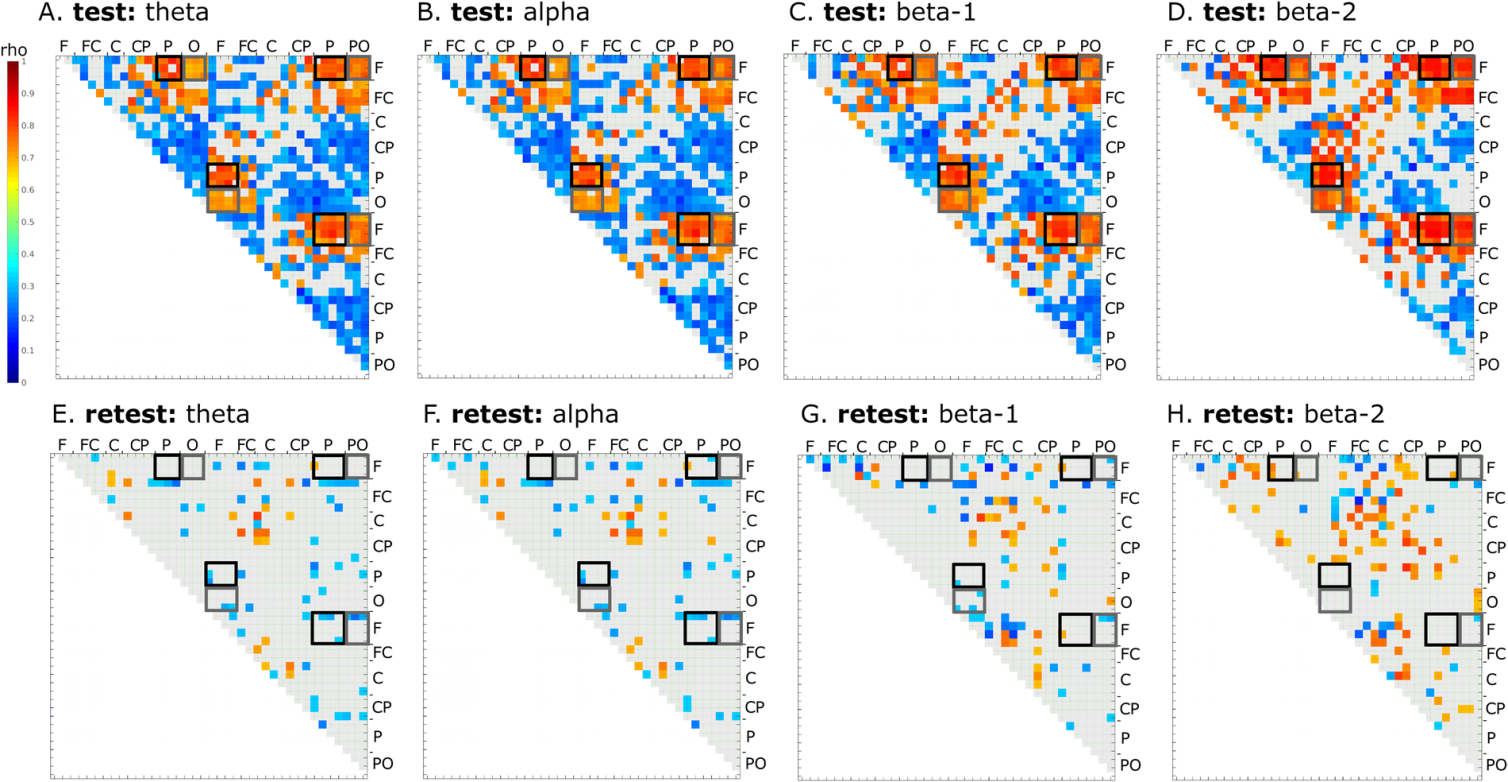
Significant correlations between gB2rest and PLVs in the visual search task (n = 33) calculated for the expectation window in test (**A–D**) and retest (**E–H**) sessions in different frequency bands. Shades of red and blue denote significant correlation strength (color bar to the left) between the two electrodes indicated next to the box outlines. Light gray squares denote lack of significance. Gray outlines denote PLVs between signals from frontal and occipital electrode sites; black outlines denote PLVs between frontal and parietal sites. All correlations significant at p < 0.05 (FDR corrected).

Analysis of EEG signals from the visual search task recorded during the test session showed a similar connectivity pattern to that obtained for the resting state. In all investigated bands, and in all windows sliding between 1700 and 0 ms before onset of target stimulus (for details see Methods) higher gB2rest values were associated with higher PLVs of the long-range frontoparietal and frontooccipital connections. Figure 3A–D shows results calculated for the 500 ms window immediately preceding onset of target stimulus. During this period, out of a possible 741 pairs of signals, the median value of significant correlations between gB2rest and PLV was 487 for theta, 494 for alpha, 507 for beta-1, and 509 for beta-2. The strongest correlations were found in the beta-2 range (Fig. 3D; see also Suppl. Inf. S2). Significant correlations between gB2rest and PLV within each of the sliding window for all investigated bands in visual search task are presented in Suppl. Inf. S3.

During the retest session (Fig. 3E–H), there were significantly fewer pairs of electrodes with significant correlations between gB2rest and PLVs than in the test session (p < 0.0079). In contrast to the test session, in all of investigated bands, only a few electrode pairs connected frontal with parietal or occipital sites.

### gB2rest versus connectivity in the shooting task

**Figure 4:**
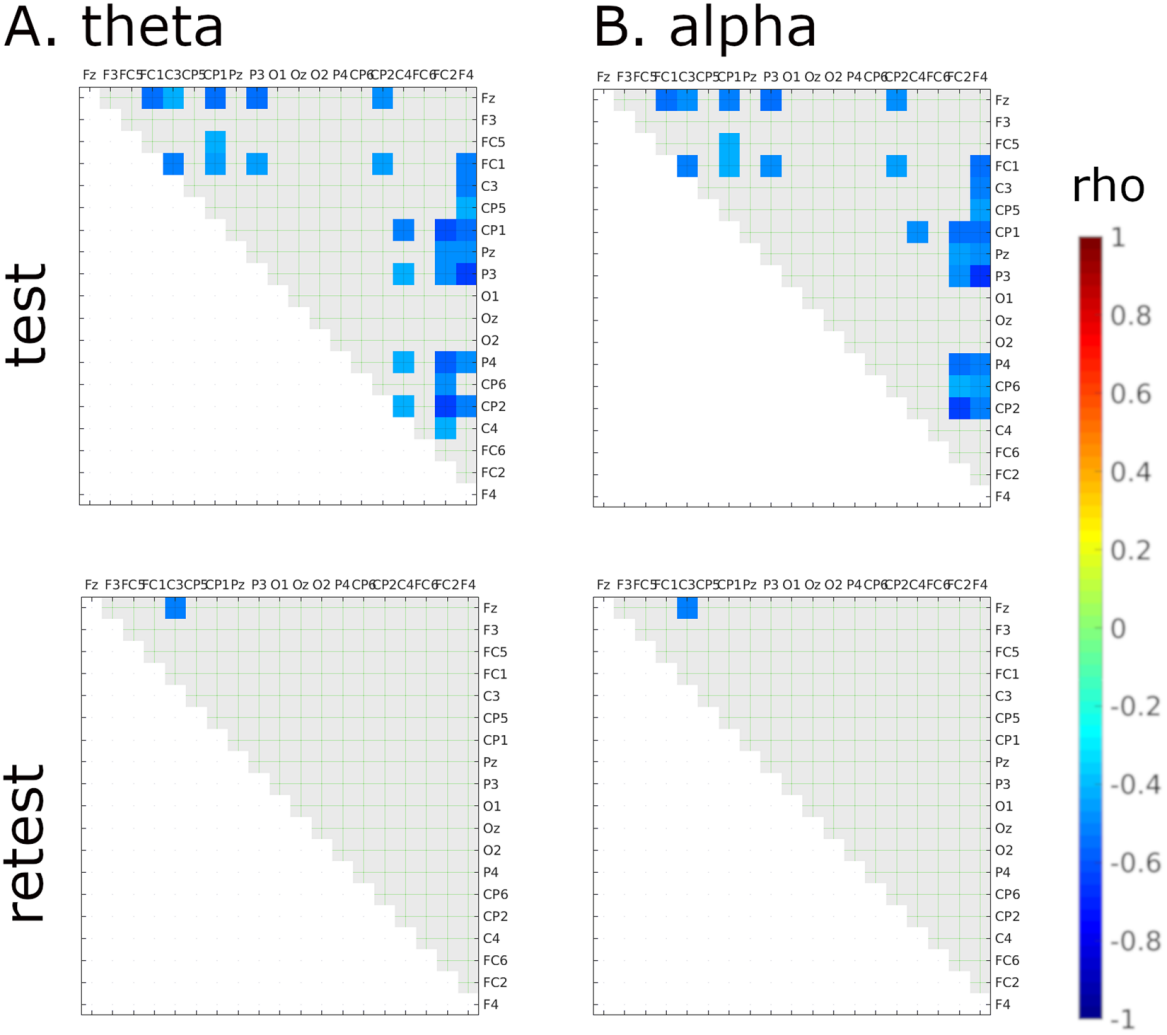
Significant correlations between gB2rest and PLVs in the shooting task (n = 23) during the expectation window in test (top row) and retest (bottom row) sessions were found only in theta (**A**) and alpha (**B**) bands. The color of individual squares denotes the correlation strength between the two electrodes that correspond to that square (uncorrected). Light gray squares denote lack of significance.

The same analysis conducted for data recorded during the test session of the shooting task revealed significant correlations between gB2rest and task-related PLVs (p < 0.05 uncorrected) only in theta (median of 35 pairs out of total 171) and alpha (median of 40 pairs out of 171 in total) bands. Correlations were found for all sliding widows (see Suppl. Inf. S4a,b for details of each sliding window and similar results for retest session). In beta-1 and beta-2 bands there were only isolated significant correlations in test and none in retest (see Suppl. Inf. S4c,d). Therefore, further analysis was focused on the theta and alpha bands. Significant negative correlations between gB2rest and PLV were found for frontal, frontocentral and central, centroparietal and parietal regions. Figure 4A,B show correlations between gB2rest and PLV for theta and alpha bands in the 500 ms window immediately preceding onset of the target stimulus.

Similar to findings for the visual search task, in the retest session of the shooting task, we found less significant correlations than in the test session (difference trending toward significance p < 0.1 for both bands). Details of the number of significant correlations in each sliding window and significance of the differences between test and retest sessions are shown in Suppl. Inf. S5.

Note that the patterns of correlations between gB2rest and PLVs in the shooting task (Fig. 4A,B) differed from those obtained in rest (Fig. 2A,B) and during the visual search task (Fig. 3A,B). These differences suggest that despite similarities in connectivity patterns between rest and laboratory-based task (computer-based visual search), the pattern registered in ecological task (shooting) differs substantially from rest (see Suppl. Inf. S6).

### Participants with low and high gB2rest differ in their capacity to modify connections strength

Theoretical analyses have shown that the stability and robustness of strong beta-phase correlations are characteristic of long, interregional connections^34–36^. Since participants with higher gB2rest were characterized by stronger resting-state PLVs (intrinsic connectivity) between frontal and parietal and between frontal and occipital sites in the beta-2 band (Figs. 2 and S2), we hypothesized that participants with stronger ties might be less prone to modification of their connections strength and therefore the smaller number of significant gB2rest-PLV correlations in the retest session observed in both tasks would result mostly from the change of connections’ strength in the participants with lower PLVs.

To test this hypothesis, we split participants into two equally numbered groups (n = 16 per group) of high and low gB2rest values. We skipped one participant with the median value (0.4273) for better separation and numerical balance between the groups. The obtained groups (called in the remaining text as ‘low gB2rest’ and ‘high gB2rest’ groups) were significantly different in their mean gB2rest values (high gB2rest group: 0.74 µV^2^ ± 0.17, low gB2rest group: 0.3 µV^2^ ± 0.08; p < 0.01, two-tailed paired t-test, n = 16).

**Figure 5:**
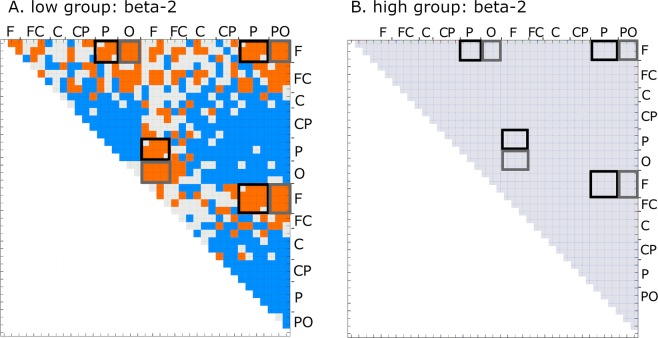
Significant (p < 0.05, FDR corrected) differences of the PLVs in the beta-2 band between retest and test sessions for the low-gB2rest group (**A**) and lack of significant differences for the high-gB2rest group (**B**) in the visual search task. Red and blue depict significant positive and negative differences, respectively, between retest and test; black outlines denote frontoparietal connections; gray outlines denote frontooccipital connections.

We next compared PLVs separately for the high and low gB2rest groups between the retest and test sessions in the visual search task using 500 ms windows, sliding between 1700 and 0 ms, before the onset of target stimulus (for details see Methods) in all investigated bands (theta, alpha, beta-1, beta-2). Significant (p < 0.05, FDR corrected) differences were only found for the low gB2rest group. Figure 5 shows representative test to retest differences in the beta-2 band obtained in the 500 ms window preceding directly target onset. Details of sliding windows analysis for the visual search task are shown in Suppl. Inf. S7.

**Figure 6:**
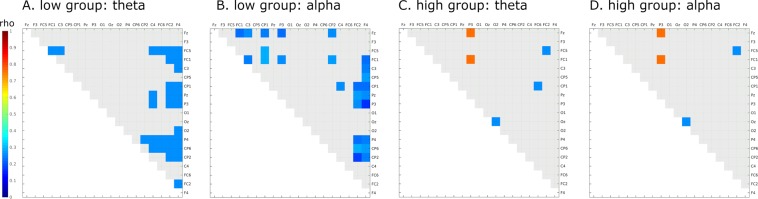
Significant (p < 0.05, uncorrected) differences in the PLVs between retest and test sessions for the low gB2rest (n = 8, A, B) and high gB2rest (n = 14, C, D) groups in the shooting task. Red and blue colors depict significant positive and negative differences, respectively, between retest and test. Light gray squares denote lack of significance. Analyses were conducted for bands that showed significant correlations between gB2rest and PLV (comp. Figure 5).

The same analysis conducted for the shooting task (high gB2rest group: n = 14, low gB2rest group n = 8) for the theta and alpha bands (which showed significant correlations between gB2rest and PLVs in test session; Fig. 4A,B) revealed (in the 500 ms window preceding directly onset of the target stimulus) over two times higher numbers of PLVs with significant differences (p < 0.05, uncorrected) between retest and test sessions for the low gB2rest group (13 out of 171 in total in each band; Fig. 6A,B) than for the high gB2rest group (5 and 6 out of 171 in total for theta and alpha bands, respectively; Fig. 6C,D). Interestingly, in the low gB2rest group, we observed a decrease in PLVs on the electrode pairs spanning the right frontal and parietal regions and connecting the frontal and frontocentral and centroparietal areas (Fig. 6A,B), while in the high gB2rest group, we observed a strengthening of strong already in resting-state frontoparietal and frontooccipital connections (Fig. 6C,D). For details of the significant group differences in the shooting task see Suppl. Inf. S8.

These observations seem to confirm our notion that participants with higher gB2rest, characterized by high intrinsic frontoparietal and frontooccipital correlations, are less predisposed to network reconfigurations.

### gB2rest and EEG activity during the anticipation time

To further elucidate the putative relationships between gB2rest and EEG activity underlying behavioral performance, we analyzed correlations between gB2rest and the power of four EEG bands (theta, alpha, beta-1 and beta-2) during the anticipation time (see Methods for definition). Analyses of the visual search data revealed significant (p < 0.05, FDR corrected) correlations between gB2rest and the alpha, beta-1 and beta-2 bands in test and between gB2rest and beta-1 and beta-2 bands in retest sessions, while in the shooting task, we found significant (p < 0.05, uncorrected) correlations only in alpha bands in the retest session. For details of the correlations between gB2rest and EEG power in the analyzed EEG bands in the 500 ms window preceding directly target onset, see Suppl. Inf. S9.

Comparison of the EEG power from test and retest sessions conducted separately for high and low gB2rest groups (for each electrode site the grand average was calculated by averaging across subjects) showed significant differences in both tasks only for the low gB2rest group. In the visual search task, we noticed an increase in the theta, beta-1 and beta-2 band power recorded by all electrodes except frontal and frontocentral in beta-1 and beta-2 bands (see Suppl. Inf. S10 for the results obtained for 500 ms window preceding directly target onset). In the shooting task, we observed a decrease in alpha power on electrodes F3, FC5 and O2 (p < 0.05, uncorrected). In the high gB2rest group of both tasks, we did not observe any difference in EEG power between retest and test for any band or for any electrode.

Thus, similar to the results of the connectivity analyses, better performing participants with lower gB2rest were more predisposed to test to retest changes than those with higher gB2rest values.

### Different task-related potentials in the high- and low-gB2rest groups

The analysis of behavioral data revealed that participants with lower gB2rest had better results (Fig. 1 and Suppl. Inf. S11). Advantage in performance was accompanied by a test to retest changes in the EEG power and PLVs of these subjects as described in the previous sections. We therefore checked whether differences could also be observed in the mean ERPs accompanying target stimuli.

**Figure 7:**
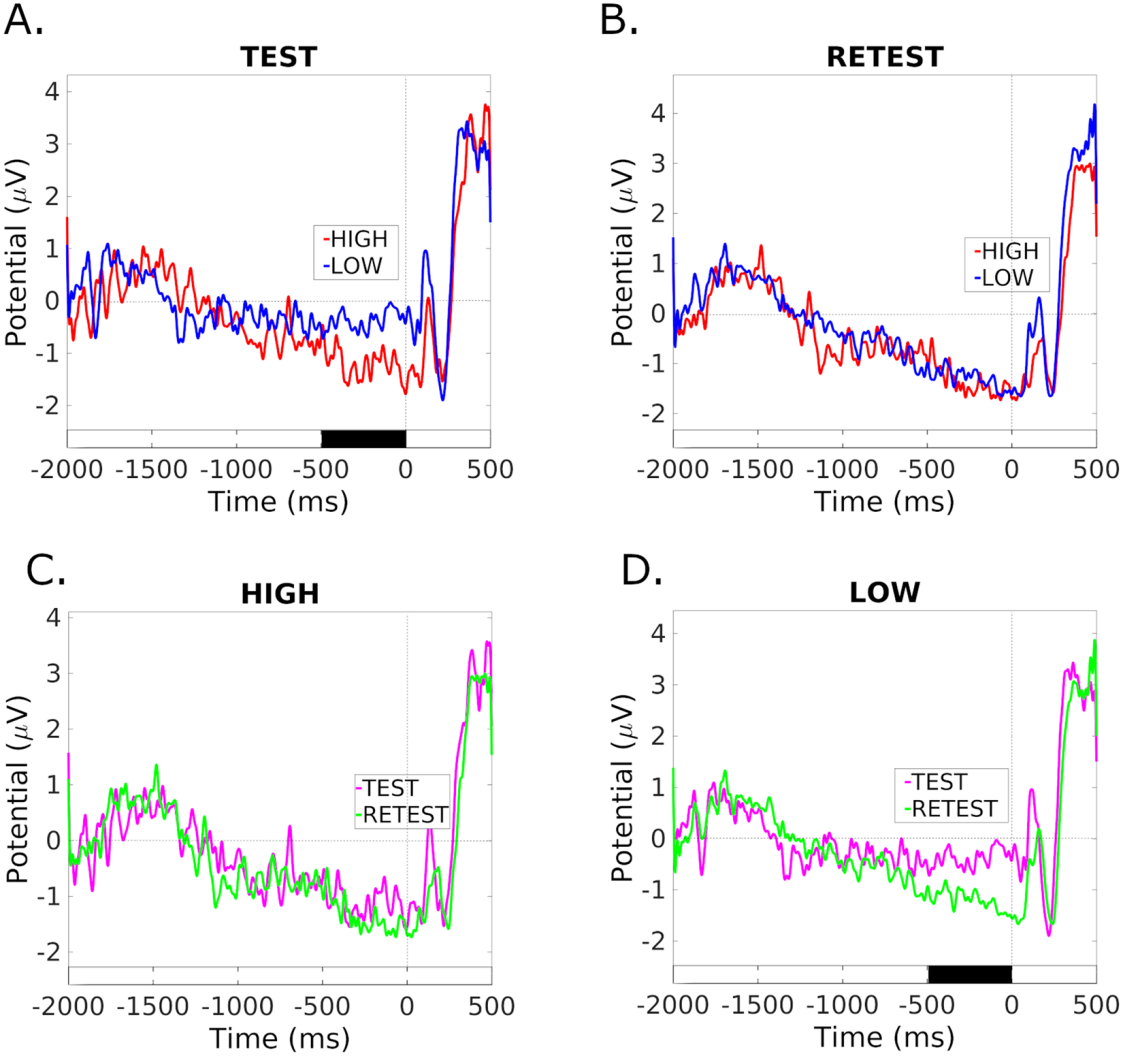
Grand average event-related potentials (ERPs) recorded from electrode Pz during the visual search task. (**A**,**B**): ERPs for the high-gB2rest (red) and low-gB2rest (blue) groups in the test (**A**) and retest (**B**) sessions. (**C**,**D**): ERPs from test (magenta) and retest (green) for the high (**C**) and low (**D**) GB2rest groups. Black bars denote the expectation window with significant differences between groups (p < 0.05, FDR corrected). Time 0 denotes target onset.

In the visual search task, to compare the ERP responses of the participants belonging to the low- and high-gB2rest groups, we used their group-averaged ERPs individually for each electrode. Consistent with the test-retest changes observed in the PLV and EEG band powers, we found alterations in the ERP courses only in the low gB2rest group. Figure 7 shows representative ERP waveforms recorded from the Pz electrode in test and retest for low and high gB2rest groups.

**Figure 8:**
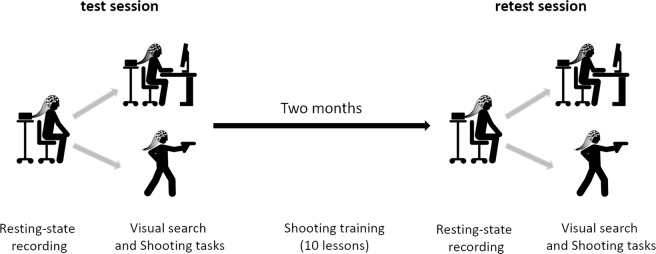
Scheme of the experiment. The experiment started with a test session comprised of an eyes open resting-state EEG recording (64 channels) preceding the visual search (64 channels) and shooting task (32 channels – wireless). After approximately two months, all tasks and recordings were repeated in a retest session. Between sessions all participants received 10 lessons of gun shooting.

During the test session, high gB2rest group ERPs were characterized by a significantly greater mean amplitude (p < 0.05; FDR corrected) of the negative wave recorded during the expectation window from centroparietal (CP1, CPz, CP2) and parietal (P1, Pz) signals relative to the low gB2rest group ERPs (see exemplary Pz potentials presented in Fig. 8(A). These differences were not found in retest (Fig. 8B). Comparison of the ERP waves between the test and retest recorded by the same electrodes separately for the high and low gB2rest groups showed increased amplitude (larger negativity) of this wave in the low gB2rest group (Fig. 8D) and no changes in the high gB2rest group (Fig. 8C), concordant with results observed in EEG power and PLV and described above. We did not find any group differences during the expectation window in the shooting task.

## Discussion

While the role of beta band activity in attentional performance is widely known, the neurophysiological mechanisms behind this relationship are still not well disclosed. As some recent studies also showed relations between EEG activity and behavioral performance^25,26^, they did not analyze the role of frontoparietal connectivity in this respect. Our data provides the first evidence that resting state beta band power is a marker of strength of frontoparietal connections. We also show that strong frontoparietal connections restrict network reconfiguration capacity and deteriorate behavioral performance. Taking together all these observations establish a link between the strength of frontoparietal and frontooccipital connections and beta band activity resulting in differences in behavioral performance as reported in numerous experiments.

### Global resting-state beta-2 power (gB2rest) predicts behavioral and physiological responses in attention tasks

Within the 2–45 Hz range of resting-state EEG, only gB2rest (power in the 22–29 Hz band averaged over all electrodes) showed stable correlation with reaction times and shooting scores in a repeated (2 months apart) visual search and shooting tasks. In both tasks, gB2rest correlated negatively with performance; higher gB2rest values were related to longer reaction times and lower shooting scores.

Investigations of the electrophysiological underpinnings of these results revealed that (i) lower gB2rest values were correlated with weaker PLVs between frontal and parietal and between frontal and occipital regions in the resting state and during task performance, (ii) during retest sessions, the number of PLVs correlating with gB2rest markedly decreased in relation to test sessions in both tasks, (iii) greater test–retest changes in the electrophysiological measures (PLV, EEG power, ERP) were observed in participants with lower gB2rest.

### Relationship among gB2rest, strength of connectivity and behavioral performance

In our experiment, better behavioral performance in test and retest sessions was observed in participants with lower gB2rest, characterized by weaker resting-state frontoparietal and frontooccipital connections. We hypothesize that weaker intrinsic long-range connectivity facilitated higher capacity of network reconfiguration in response to task demand. This notion seems to be well grounded in theoretical investigations. Strong long-range connections were found to be highly stable^34–36^, less prone to disturbance and less energetically demanding^34,36^. Stronger phase correlations were also postulated to lower network complexity, resulting in less flexible processing^37,38^ and worse behavioral performance^39^.

Although these theoretical considerations and their applications to neuronal networks are well established, they are still not sufficiently documented by experimental investigations. In one of few studies devoted to the role of weak resting-state connectivity, Santarnecchi and colleagues^40^ found, in their fMRI experiment, positive correlations between cognitive abilities (measured with the Wechsler Abbreviated Scale of Intelligence) and global resting-state network efficiency of weak connectivity. More detailed analyses revealed that these connections were linking distant brain lobes between and within hemispheres. These findings are concordant with our results, as we found that better performing participants were characterized at rest by weaker long-range connectivity between frontal and occipital as well as between frontal and parietal regions of both hemispheres (Fig. 3). Santarnecchi and colleagues^40^ suggested that stronger connectivity, constituting a stable framework of the functional architecture, assured a high degree of stability, whereas individual variability related to high-order cognition was better explained by weak ties. In those authors’ opinion, increased information transfer between brain regions connected through weak long-range connections rationalized to a large extent better cognitive performance, which could also match our data. A negative relationship between the strength of task-related connectivity in the low beta signal (~15 Hz) measured before target onset and cognitive performance was also found in MEG investigations of the attentional blink^28^. These observations are also concordant with our results showing that participants with a higher capacity for network reconfigurations performed better than those with a lower capacity.

Strong phase couplings of frontal and parietal regions characterized by high stability and robustness for interactions^34,41^ could also explain negligible differences in the strength of connections between test and retest sessions observed in visual search task in the participants with higher gB2rest values (Fig. 5B). These negligible connectivity changes in the high gB2rest participants in conjunction with the increased strengths of the long-range ties in the participants with lower gB2rest values (Fig. 5A) could lead to reduced correlation between gB2rest and PLVs as shown in the lower panels of the Figs. 4 and 5. At the same time, strengthening of the frontoparietal connections in the participants of lower gB2rest group (Fig. 5A) observed in visual search task may indicate learning processes leading to better behavioral performance of this group in test and retest.

Support for the notion that test to retest connectivity changes are induced by learning processes may be inferred from an experiment recording extracellular potentials in gerbils^42^ where a positive effect of strengthening of both bottom-up inputs and top-down modulation on performance in perceptual learning task was found. Similarly, fMRI results from Cole and colleagues^43^ suggest that flexible alterations of functional connectivity within frontoparietal networks enables highly adaptive behavior of first-trial task learning.

Co-occurrence of test to retest changes in all investigated electrophysiological measures (ERP, EEG powers and PLV) may suggest their interdependence. Contribution of different brain sources to the waves of elicited ERP components^44–46^ and theoretical investigations on interdependence between strength of connectivity and EEG spectral power^47,48^ seem to confirm this notion. For example, direct investigations of the functional connectivity and ERP components elicited by oddball paradigm revealed functional connection between central and frontal regions during P300 waves^44^. Also, neural mass models suggest that interregional coupling is one of critical determinants of the EEG spectrum^48,49^. Since in our experiment both ERP and spectral power changes were related to strength of the phase locking value between frontal and parietal regions we posit that changes in functional connectivity (possibly resulting from learning processes) are one of the main sources of alterations of spectral power and ERP components accompanying cognitive tasks.

### Differences between the visual search and shooting tasks

While the results of studies by Santarnecchi and colleagues^40^ and Gross and colleagues^28^ are consistent with those of visual search tasks performed in our laboratory, all of them differ from the outcome of our shooting task. The correlations between gB2rest and PLVs in the shooting task were different from those observed during the visual search task. Positive correlations between gB2rest and the strength of fronto-parieto-occipital PLVs found in the resting state and in test sessions of our visual search task were observed only in a few pairs of signals in the theta and alpha bands on retesting, while, on testing, we found negative correlations between gB2rest and right frontoparietal connections (probably related to response inhibition^50–52^). Additionally, the group differences found in the ERP during the expectation window in the visual search task were not present in the shooting task. We speculate that in the test session of the shooting task, the high variability of the EEG signal induced by the complexity of visuomotor coordination masked the existing positive correlations between gB2rest and the strength of frontoparietal connections. We posit that the shooting practice, which took place between recording sessions, allowed subjects to gradually master the task, which reduced signal variability on retesting and uncovered existing correlations between gB2rest and the strength of frontoparietal connections (Fig. 6C,D).

Despite the above caveats, we found general similarities between computerized visual search and ecological shooting tasks. First, we found a similar relationship between strong frontoparietal resting-state connections and poor behavioral performance in visual search and shooting tasks. Second, the test–retest differences in EEG power in both tasks and in ERP in the case of the visual search task seemed to follow changes in the PLVs, suggesting their network origin. Third, the strength of the frontoparietal resting-state connections was closely related to global beta-2 resting-state activity.

### The role of the beta frequency band in attention tasks

The positive correlations between resting-state PLVs and the power of the beta-2 band (gB2rest), as found in our experiment, confirm the notion that beta band activity plays an essential role in attentional processes, as postulated long ago in our laboratory^53–55^. The top-down attention pathway, which spans several frontal and parietal regions^56–58^ and is associated with attentional behavior in animals^53–55,59^ and humans^2,3,29,32,57,60–62^, seem to be active in the resting state, as confirmed by our present experiment.

Thus, we posit that the shorter reaction times and higher shooting scores observed in both tasks and both experimental sessions in the participants with lower gB2rest were related to weaker intrinsic frontoparietal and frontooccipital connections, resulting in a greater capacity for network reconfiguration. In contrast, participants with higher gB2rest, showing stronger intrinsic long-range connections, could benefit from cost-effective strategies^34,36^.

### Innate origins of EEG properties and their consequences

The relations between connection strength and capacity for network reconfiguration and subsequent behavioral performance is one of the resting-state properties which have been proposed to be genetically determined^63^. Also other properties of the EEG signal such as power, connectivity or network organizations have been found to depend on inherited traits^64–68^. Thus, different strength of resting-state frontoparietal connections characterizing individuals from high and low gB2rest experimental groups may result in different realization of the same behavioral or cognitive tasks and explain ambiguous results in similar experiments.

### Limitations and future work

Our current study demonstrated that the shorter reaction times and higher shooting scores observed in both tasks and both experimental sessions in the participants with lower gB2rest were related to weaker intrinsic frontoparietal and frontooccipital connections. These conclusions were drawn based on the experiment conducted in the laboratory and in the shooting range on the unisex group of participants. This approach, however, resulted in relatively high exclusion rate of the EEG signal collected during shooting exercise, due to movement artifacts, which lowered statistical power of the analyses and ignores the possible sex differences. Future work has to examine if current results could be replicated with the group of participants balanced for the sex and with field task adjusted to account for high exclusion rate of the EEG signal.

## Methods

### Participants

We examined 36 healthy adult males recruited by announcements at local universities. The exclusion criteria included neurological disorders, brain injury, current use of analgesic medication, substance abuse or dependence and mental disorders. We also excluded women from the experiment due to possible EEG changes associated with the menstrual cycle^69–72^. All participants were right-handed and had normal or corrected-to-normal vision, and the mean ± standard deviation of their age was 21.97 ± 1.88 years.

The experimental procedures were approved by the local bioethics committee at Military University of Technology in Warsaw. All participants gave their written informed consent to participate in the experiment in accordance with the WMA Declaration of Helsinki – Ethical Principles for Medical Research Involving Human Subjects. All experiments were performed in accordance with all relevant guidelines and regulations.

### Experimental procedures

There were two behavioral tasks performed by all participants: a visual search attention task and a separate gun-shooting task. Each task was performed twice, with an interval of approximately two months between the first (‘test’) and second (‘retest’) sessions (Fig. 8).

#### Visual search attention task

**Figure 9:**
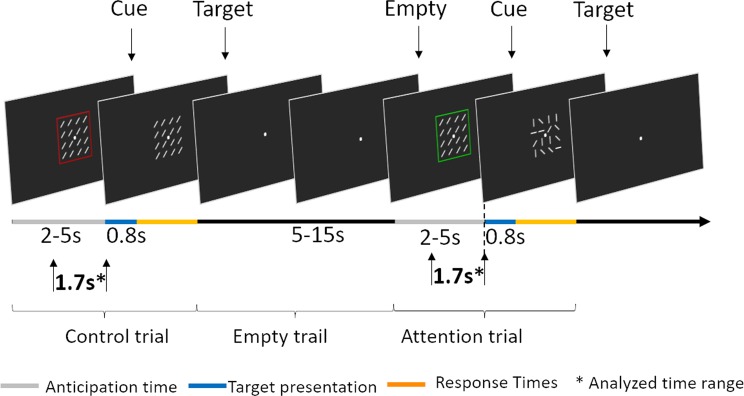
Visual search experiment set-up. Participants were presented with the control trials, attention trials and empty trials. During control trials, participants were asked to indicate on the target matrix the direction of the identically oriented bars. In the attention trials, participants were expected to confirm whether the bar with orientation specified on the cue matrix was present among differently oriented bars on the target. Empty trials were devoid of matrix stimulus. Cue presentation (anticipation time) lasted 2–5 s and ended with presentation of the target matrix for 800 ms. The data used for analyses were extracted from the 500 ms sliding windows (300 ms overlap) moving over 1700 ms preceding the onset of target matrices (expectation windows) marked by arrows and stars.

This computerized task (Fig. 9) aimed to activate endogenous (top-down) attention processes and was based on the test previously described by Buschman and Miller^56^. Participants were asked to search a target matrix for a white bar with the orientation indicated by the cue. The task consisted of attention and control trials. The type of trial was indicated by the color of the frame surrounding the stimulus matrices: green for attention trials or red for control trials. Each trial started with the presentation of a fixation point, followed by the cue and target stimuli, each consisting of 16 small white bars (arranged in a 4 × 4 matrix) on a black background. All white bars in a cue matrix were identically oriented in one of four possible orientations: vertical, horizontal, or tilted 45° to the left or right. The target stimulus in the attention trials comprised 16 differently oriented white bars that included (or did not include) one matching the orientation of the cue. In the attention trials, the participant’s task was to indicate whether the bar whose orientation matched that of the cue was present in the target matrix by pressing a key with the right hand for a match or with the left hand for no match. In the control trials, all 16 bars of the cue and target had the same orientation (tilted left or right), eliminating to a large extent the attentional component, and the participant’s task was to indicate the orientation of the bars with the corresponding (left or right) hand.

Participants were allowed a time window of 1800 ms to respond (800 ms during presentation of the target matrix and 1000 ms immediately afterward). To avoid temporal conditioning, the cue duration (‘anticipation time’) was randomly assigned (2, 3, 4 or 5 s), with the numbers of each cue length equally distributed among attention and control trials. However, for the EEG analysis, we used the signal from the last two seconds preceding the target (matching the shortest cue duration). The onset of the target immediately followed the offset of the cue. To introduce variance among the intertrial intervals, we distributed additional, ‘empty trials’ of 5, 10 or 15 s length with semirandom order, for which only a fixation point was presented. In total, the task consisted of 120 trials (48 attention, 48 control and 24 ‘empty trials’ devoid of stimuli) and lasted 12 minutes.

The experimental task was preceded by instructions and short practice (10 trials) for each participant. The behavioral data from the attention task consisted of reaction times, measured as the time from target onset to a button press, and accuracy, calculated as the percent of correct responses among the total number of trials. Both measures were accompanied by ongoing EEG imaging. The visual search task was repeated twice at a two-month interval (in ‘test’ and ‘retest’ sessions).

#### Shooting task

The second task involved a shooting test with a pneumatic gun. Shooting was chosen as the field test for its controlled yet natural environment, and electronic results measurement assured the similarity of conditions and objectivity of comparisons. The first (‘test’) shooting session was followed by regular sport shooting practice finalized after two months with a ‘retest’ session. Both test and retest shooting sessions were conducted after the complementary visual search tests and were accompanied by ongoing EEG recording. Both shooting tests and shooting training were conducted by a professional coach and took place in an indoor shooting range at a distance of six meters with a Feinwerkbau 65 the pneumatic gun (Oberndorf, Germany) with a caliber of 4.5 mm. The shooting target was scaled proportionally to the distance according to the regulations for the Olympic Games. The test comprised 40 shots to be completed within 1 hour, and the single shot time was not limited. The maximum score for each shot was 10 points (so the best score equaled 400 points). The shooting tests were conducted using a SCATT Shooting System (SCATT Electronics LLC, Russia), which registered shooting scores and times. To mark trigger pulling in the EEG recording, we used a highly sensitive microphone with an amplifier tuned to the sound of a trigger while shooting. The signal was then transferred to AUX inputs in the amplifier and registered as the shot marker.

### Electrophysiological data

For the analyses of task related and attention-oriented EEG signal we used last two-seconds of each trial, matching the shortest duration of the cue. To exclude EEG signal contamination from preceding responses to visual stimuli and artifacts from gun trigger movement, we excluded from our analyses first 300 ms of the two seconds period leaving for analyses 1.7 s-long expectation period immediately preceding target onsets in the visual search task (Fig. 1) and trigger pulling in the shooting task. These periods encompassed increased visual attention, including both top-down and bottom-up attention components (see Fig. 9). To account for dynamics of the EEG signal, and to allow statistical comparisons, analyses were conducted using a 500 ms sliding window with 300 ms overlap. The length of the window was chosen to ensure the sensitivity of EEG synchrony measures which was found to be poor when performed on signals below 400 ms^73^.

#### Recording and preprocessing

The EEG recording accompanying the visual search task started with a 2-minute resting-state EEG registration with eyes open. The visual search task datasets were recorded with 64 Ag/AgCl electrodes (Quick Amp; Brain Products GmbH) with a reference electrode positioned at fixed FCz or Cz sites (for technical reasons, the original cap with the FCz reference electrode had to be replaced after the first 10 participants, and the remaining 26 were examined with a new cap that had the reference electrode positioned at Cz). After all recordings were completed, the raw signals of the Cz reference electrode were rereferenced to FCz with the Brain Analyzer software. Since the calculated differences in ERPs and phase locking values between signals with FCz and Cz references were found to be negligible (not shown) we decided to use FCz site for reference due to the fact that this data were already preprocessed and used for other analyses. Nine electrodes which were differently positioned or used as the reference in the two systems were excluded from further analyses (Iz, A1, A2, FPz, FCz, T9, T10, TP9 and TP10) leaving in total 55 electrodes.

During the shooting task, EEG was recorded with 32 Ag/AgCl electrodes (Quick Amp; Brain Products GmbH) positioned in an ActiCap (Brain Products GmbH) with a reference electrode positioned at the FCz site connected to the amplifier via a wireless transmitter (Move, Brain Products GmbH).

The recording electrodes were positioned according to the extended 10–20 system and digitized at a sampling rate of 500 Hz. The impedance for all electrodes was maintained below 10 KΩ. The ground electrode was placed at the FPz position in both systems. Preprocessing was conducted in the open source EEGLAB toolbox^74^ and custom MATLAB 2016a scripts (The MathWorks, Inc.). The preprocessing pipeline included 0.5–70 Hz bandpass filtering, baseline correction, exclusion of 1 s data segments containing artifacts (EEGLAB, autorej function) and independent component analysis (ICA) removal of eye movement and muscle artifacts. If any of the four data sets (two resting-states and test and retest of each task) recorded from a given participant needed to be removed due to excessive artifacts or missing signal (i.e., if the number of remaining trials was less than 50%), that data was excluded from the analysis. Similarly, we removed data from participants if the signal from their resting-state recording that remained after cleaning had a total length of less than 90 s. In total, the data sets of 3 participants in the visual search task and 13 in the shooting task were removed, leaving 33 and 23 participants in visual search and shooting task, respectively, for further analyses. Next, the signals from the most contaminated electrodes (Fp1, Fp2, AF7, AF3, AF8, AF4, F7, F8, FT7, FT8, FT9, FT10, T7, T8, TP7, TP8, P7, P8, PO7, PO8, PO9, PO10 for 64 electrodes system and Fp1, Fp2, F7, F8, FT9, FT10, T7, T8, TP9, TP10, P7, P8, for 32 electrode system) were excluded from the analyses in all participants, leaving signals from 39 out of 55 electrodes in 64 channel system and 19 out of 32 electrodes in 32 channel system (in visual search and shooting task, respectively) per participant for the subsequent analyses (comp. Fig. S7). This relatively high exclusion rate was due to movement artifacts, typical for tasks in the sporting domain (e.g.^75^).

#### EEG connectivity analysis

As a proxy for interregional cortical connectivity, we used the phase-locking value (PLV) calculated for 500 ms windows with 300 ms overlap sliding between 1700 and 0 ms before onset of target stimulus. Originally defined as a measure of synchrony^76^ PLV is commonly used to estimate connectivity in EEG/MEG studies^77,78^.

We used the PLV measure because it does not depend on spectral power of the recorded signals. The mean spectral powers recorded from our subjects differed significantly, which could bias source reconstruction or connectivity estimated by other measures based on amplitude values^76^. Moreover, the PLV was found to be robust to noisy signal^79^ and our recordings collected during shooting task were highly contaminated by movement and muscle artifacts. Finally, the PLV provides also more sensitive connectivity measure than phase lag index^80^ and it is recommended for investigations driven by hypothesis^79^. The possibility of potential confounds coming from volume conduction (as PLV is more prone to these confounds that phase lag index) was self-controlled in our paradigm as the connectivity values were compared between groups of the same subjects (in test-retest or rest-task comparisons; see chapter 4.6 below).

The PLV estimates phase covariance between two signals by separating the phase and amplitude components. To compute PLV in a given frequency range, we filtered the EEG data using a two-sided finite impulse response filter and then subjected them to a Hilbert transform for computation of the instantaneous amplitude and phase. Only the phase component was used for PLV computation. The normalized difference between phase time courses of signals from two electrodes quantified locking between the phases. The PLVs were calculated during the expectation window for all pairs of electrodes, first individually for each participant/session and then as group averages. Note that the PLV pattern of all cooperating/coordinated signal pairs does not contain the direction of information flows.

#### Spectral power and ERP analysis

The power spectrum of four canonical EEG bands (theta, 4–7 Hz; alpha, 8–12 Hz; beta-1, 13–21 Hz and beta-2, 22–29 Hz) was calculated individually for each electrode (averaged over trials) for each sliding window.

For the ERP analysis, epochs lasting 2.5 s each, starting 2 s before target onset (matching the shortest cue duration), were extracted from signals collected from each electrode. The mean potential value obtained from resting-state data preceding a given session (test or retest) was used for ERP baseline correction during that session. Group-averaged ERPs (using the eegplot function from the EEGLAB toolbox^73^); were drawn and inspected for waveform differences. To verify significant waveform differences during the expectation window, we performed, for each electrode, two-tailed two-sample t-tests comparing averaged potentials within each sliding window and corrected the results for multiple comparisons using the false discovery rate (FDR) method^81^.

### Definition of a behaviorally relevant frequency band

We used the resting-state data from the first recording session (test) to search whether spontaneous activity of any of the frequency bands could be behaviorally relevant, i.e., predictive of the behavioral results of attention tests and/or the features of EEG recorded during task performance. The resting-state signals from all electrodes were analyzed using a fast Fourier transform (FFT) moving average method (window width 1024 ms, 750 ms overlap). The resulting FFTs were averaged within seven regions of interest (ROIs) along the anterior–posterior axis (frontal, frontocentral, central, centroparietal, parietal, parietooccipital, occipital, as in^82^; see Supplementary Information S13) for each participant. Next, we calculated the mean power of narrow frequency bands of each ROI (3 Hz window with a 1 Hz overlap moving over the range from 2 to 45 Hz). After checking for outliers (none of the results was outside the limit of 3 standard deviations defining outliers), the mean power was correlated with the average reaction times and accuracy values of the control and attention trials in the visual search task and with shooting scores.

Significant correlations were found only for attention trials between reaction times/shooting scores and a power of most of the 3 Hz windows in the range of 14–45 Hz. Since these correlations applied to all ROIs, we decided to average the signals from all 39 electrodes to obtain a global resting-state power spectrum in a given frequency window. In the last step, adjacent windows showing significant correlations with parameters of both behavioral tasks (reaction time in visual search and shooting scores) were clustered, and the power within the defined frequency range was extracted as a mean of all its bands and correlated with behavioral results obtained by the participants in the first (test) and second (retest) sessions.

### Statistical analyses

Data were tested for normality by the Kolmogorov–Smirnov test and checked for the presence of outliers. All correlations of behavioral and electrophysiological data were performed using Spearman’s correlation except for similarity calculation. Similarity analyses were conducted using the Pearson correlation coefficient. Comparison of the group means was conducted using two-tailed two-sample t-tests or Wilcoxon for paired data. All results were corrected for false positives using FDR adjustment were appropriate unless otherwise stated.

### Mitigation of possible volume conduction/impedance confounds

The high and low groups were defined on the basis of averaged EEG powers. Some of the obtained power and connectivity differences between the groups could be attributed to differences in impedance or volume conduction^78,83^. To preclude possible effects of impedance and volume conduction on the final results we compared averaged impedance of electrodes used for definition of behaviorally relevant bands for high and low beta-2 groups and calculated correlations and ANOVA analyses for reaction times and corresponding ERP/FFT/PLV data. For data which was less contaminated by movement and muscle activity (recorded during visual search experiment) we also repeated connectivity analyses with use of the phase lag index. The results of the analyses are provided in Supplementary Materials S12 and S14.

## Supplementary information

Supplementary information.

## Data Availability

The datasets generated during and/or analyzed during the current study are available from the corresponding author on reasonable request.

## References

[1] Busch NA, Dubois J, VanRullen R (2009). The Phase of Ongoing EEG Oscillations Predicts Visual Perception. Journal of Neuroscience.

[2] Gola M, Kamiński J, Brzezicka A, Wróbel A (2012). Beta band oscillations as a correlate of alertness - Changes in aging. International Journal of Psychophysiology.

[3] Kamiński J, Brzezicka A, Gola M, Wróbel A (2012). Beta band oscillations engagement in human alertness process. International Journal of Psychophysiology.

[4] Molle M, Marshall L, Wolf B, Fehm H, Born J (1999). EEG complexity and performance measures of creative thinking. Psychophysiology.

[5] Touryan J, Lance BJ, Kerick SE, Ries AJ, McDowell K (1999). 2015. Common EEG features for behavioral estimation in disparate, real-world tasks. Biological Psychology.

[6] Cordes D (2000). Mapping Functionally Related Regions of Brain with Functional Connectivity MR Imaging. American Journal of Neuroradiology.

[7] Hampson M, Peterson BS, Skudlarski P, Gatenby JC, Gore JC (2002). Detection of functional connectivity using temporal correlations in MR images. Hum. Brain Mapp..

[8] Lowe MJ, Mock BJ, Sorenson JA (1998). Functional Connectivity in Single and Multislice Echoplanar Imaging Using Resting-State Fluctuations. NeuroImage.

[9] De Luca M, Smith S, De Stefano N, Federico A, Matthews PM (2005). Blood oxygenation level dependent contrast resting state networks are relevant to functional activity in the neocortical sensorimotor system. Experimental Brain Research.

[10] Fox MD (2005). The human brain is intrinsically organized into dynamic, anticorrelated functional networks. Proceedings of the National Academy of Sciences.

[11] Greicius MD, Krasnow B, Reiss AL, Menon V (2003). Functional connectivity in the resting brain: A network analysis of the default mode hypothesis. Proceedings of the National Academy of Sciences.

[12] Di X, Biswal BB (2015). Dynamic brain functional connectivity modulated by resting-state networks. Brain Structure and Function.

[13] Hearne LJ, Cocchi L, Zalesky A, Mattingley JB (2017). Reconfiguration of Brain Network Architectures between Resting-State and Complexity-Dependent Cognitive Reasoning. Journal of Neuroscience.

[14] Spadone S (2015). Reorganization of RSNs during attention. Proceedings of the National Academy of Sciences.

[15] Kelly AMC, Uddin LQ, Biswal BB, Castellanos FX, Milham MP (2008). Competition between functional brain networks mediates behavioral variability. NeuroImage.

[16] Hampson M, Driesen NR, Skudlarski P, Gore JC, Constable RT (2006). Brain Connectivity Related to Working Memory Performance. Journal of Neuroscience.

[17] Gordon EM, Stollstorff M, Vaidya CJ (2012). Using spatial multiple regression to identify intrinsic connectivity networks involved in working memory performance. Hum. Brain Mapp..

[18] Cole MW, Yarkoni T, Repovš G, Anticevic A, Braver TS (2012). Global Connectivity of Prefrontal Cortex Predicts Cognitive Control and Intelligence. Journal of Neuroscience.

[19] Mennes M (2010). Inter-individual differences in resting-state functional connectivity predict task-induced BOLD activity. NeuroImage.

[20] Sala-Llonch, R., *et al* Dynamic Functional Reorganizations and Relationship with Working Memory Performance in Healthy Aging. Frontiers in Human Neuroscience 6–152, 10.3389/fnhum.2012.00152 (2012). 10.3389/fnhum.2012.00152 PMC3369258 22701409

[21] Fox, M. D. & Raichle, M. E. Spontaneous fluctuations in brain activity observed with functional magnetic resonance imaging. *Nat. Rev. Neurosci*. 8, 10.1038/nrn2201 (2007). 10.1038/nrn2201 17704812

[22] Sadaghiani S (2010). Intrinsic Connectivity Networks, Alpha Oscillations, and Tonic Alertness: A Simultaneous Electroencephalography/Functional Magnetic Resonance Imaging Study. Journal of Neuroscience.

[23] Irrmischer M, Poil SS, Mansvelder HD, Intra FS, Linkenkaer-Hansen K (2017). Strong long-range temporal correlations of beta/gamma oscillations are associated with poor sustained visual attention performance. European Journal of Neuroscience..

[24] MacLean MH, Arnell KM, Cote KA (2012). Resting EEG in alpha and beta bands predicts individual differences in attentional blink magnitude. Brain and Cognition.

[25] Iemi L, Chaumon M, Crouzet SM, Busch NA (2016). Spontaneous Neural Oscillations Bias Perception by Modulating Baseline Excitability. Journal of Neuroscience.

[26] van Son D (2019). Electroencephalography theta/beta ratio covaries with mind wandering and functional connectivity in the executive control network. Ann. N.Y. Acad. Sci..

[27] Buschman TJ, Miller EK (2007). Top-down versus bottom-up control of attention in the prefrontal and posterior parietal cortices. Science.

[28] Gross J (2004). Modulation of long-range neural synchrony reflects temporal limitations of visual attention in humans. Proceedings of the National Academy of Sciences.

[29] Wang X (2010). Neurophysiological and Computational Principles of Cortical Rhythms in Cognition. Physiological Reviews.

[30] Tambini A, Ketz N, Davachi L (2010). Enhanced Brain Correlations during Rest Are Related to Memory for Recent Experiences. Neuron.

[31] Cole MW, Bassett DS, Power JD, Braver TS, Petersen SE (2014). Intrinsic and task-evoked network architectures of the human brain. Neuron.

[32] Cole MW, Ito T, Bassett DS, Schultz DH (2016). Activity flow over resting-state networks shapes cognitive task activations. Nature Neuroscience.

[33] Xu. J (2016). Large-scale functional network overlap is a general property of brain functional organization: Reconciling inconsistent fMRI findings from general-linear-model-based analyses. Neuroscience and Biobehavioral Reviews.

[34] Ermentrout GB, Kopell N (1990). Oscillator Death in Systems of Coupled Neural Oscillators. SIAM Journal on Applied Mathematics.

[35] Kopell N, Ermentrout B, Whittington MA, Traub R (2000). Gamma rhythms and beta rhythms have different synchronization properties. Proceedings of the National Academy of Sciences of the United States of America.

[36] Chandrasekaran L, Achuthan S, Canavier CC (2010). Stability of two cluster solutions in pulse coupled networks. Journal of Computational Neuroscience.

[37] Goldberger AL (2002). Fractal dynamics in physiology: alterations with disease and aging. Proc Natl Acad Sci USA.

[38] Zappasodi F (2014). Fractal dimension of EEG activity senses neuronal impairment in acute stroke. PLoS ONE.

[39] Tzagarakis C, Thompson A, Rogers RD, Pellizzer G (2019). The Degree of Modulation of Beta Band Activity During Motor Planning Is Related to Trait Impulsivity. Frontiers in Integrative Neuroscience.

[40] Santarnecchi E, Galli G, Polizzotto NR, Rossi A, Rossi S (2014). Efficiency of weak brain connections support general cognitive functioning. Human Brain Mapping.

[41] Gallos LK, Sigman M, Makse HA (2012). The conundrum of functional brain networks: Small-world efficiency or fractal modularity. Frontiers in Physiology.

[42] Caras ML, Sanes DH (2017). Top-down modulation of sensory cortex gates perceptual learning. Proc Natl Acad Sci USA.

[43] Cole, M. W., *et al* Multi-task connectivity reveals flexible hubs for adaptive task control. *Nat. Neurosci*., 10.1038/nn.3470 (2013). 10.1038/nn.3470 PMC3758404 23892552

[44] Bledowski C (2004). Localizing P300 Generators in Visual Target and Distractor Processing: A Combined Event-Related Potential and Functional Magnetic Resonance Imaging Study. Journal of Neuroscience.

[45] Yamazaki T (2000). Multiple equivalent current dipole source localization of visual event-related potentials during oddball paradigm with motor response. Brain Topogr.

[46] Yamazaki T, Kamijo K, Kiyuna T, Takaki Y, Kuroiwa Y (2001). Multiple dipole analysis of visual event-related potentials during oddball paradigm with silent counting. Brain Topogr.

[47] Karamzadeh N, Medvedev A, Azari A, Gandjbakhche A, Najafizadeh L (2013). Capturing dynamic patterns of task-based functional connectivity with EEG. NeuroImage.

[48] Zavaglia M, Astolfi L, Babiloni F, Ursino M (2008). The Effect of Connectivity on EEG Rhythms, Power Spectral Density and Coherence Among Coupled Neural Populations: Analysis With a Neural Mass Model. IEEE Trans. Biomed. Eng..

[49] David O, Friston KJ (2003). A neural mass model for MEG/EEG. NeuroImage.

[50] Aron, A. R., Robbins, T. W. & Poldrack, R. A. Inhibition and the right inferior frontal cortex: One decade on. *Trends in Cognitive Sciences*, 10.1016/j.tics.2013.12.003 (2014). 10.1016/j.tics.2013.12.003 24440116

[51] Fassbender C (2006). The Role of a Right Fronto-Parietal Network in Cognitive Control. Journal of Psychophysiology.

[52] Rubia K, Smith AB, Brammer MJ, Taylor E (2003). Right inferior prefrontal cortex mediates response inhibition while mesial prefrontal cortex is responsible for error detection. NeuroImage.

[53] Bekisz M, Wróbel A (1993). 20 Hz rhythm of activity in visual system of perceiving cat. Acta Neurobiologiae Experimentalis.

[54] Wróbel A, Ghazaryan A, Bekisz M, Bogdan W, Kaminski J (2007). Two Streams of Attention-Dependent Activity in the Striate Recipient Zone of Cat’s Lateral Posterior-Pulvinar Complex. Journal of Neuroscience.

[55] Wróbel A (2000). Beta activity: A carrier for visual attention. Acta Neurobiologiae Experimentalis..

[56] Buschman TJ, Miller EK (2007). Top-Down Versus Bottom-Up Control of Attention in the Prefrontal and Posterior Parietal Cortices. Science.

[57] Hwang K, Shine JM, D’Esposito M (2019). Frontoparietal Activity Interacts With Task-Evoked Changes in Functional Connectivity. Cerebral Cortex.

[58] Wang L (2017). Beta-band functional connectivity influences audiovisual integration in older age: An EEG study. Frontiers in Aging. Neuroscience..

[59] Bekisz M (2016). The primary visual cortex is differentially modulated by stimulus-driven and top-down attention. PLoS ONE.

[60] Bastos AM (2015). Visual areas exert feedforward and feedback influences through distinct frequency channels. Neuron.

[61] Saalmann YB (2007). Neural mechanisms of visual attention: How top-down feedback highlights relevant locations. Science.

[62] Stein van A, Chiang C, König P (2000). Top-down processing mediated by interareal synchronization. Proceedings of the National Academy of Sciences of the United States of America.

[63] Vuoksimaa E (2017). Heritability of white matter microstructure in late middle age: A twin study of tract‐based fractional anisotropy and absolute diffusivity indices. Hum. Brain Mapp..

[64] Glahn DC (2010). Genetic control over the resting brain. Proceedings of the National Academy of Sciences.

[65] Malone SM (2014). Heritability and molecular-genetic basis of resting EEG activity: A genome-wide association study. Psychophysiology.

[66] Posthuma D (2005). Genetic components of functional connectivity in the brain: The heritability of synchronization likelihood. Human Brain Mapping.

[67] Smit DJA, Stam CJ, Posthuma D, Boomsma DI, De Geus EJC (2008). Heritability of “small-world” networks in the brain: A graph theoretical analysis of resting-state EEG functional connectivity. Human Brain Mapping.

[68] Schutte NM (2013). Heritability of resting state EEG functional connectivity patterns. Twin Research and Human Genetics.

[69] Creutzfeldt OD (1976). EEG changes during spontaneous and controlled menstrual cycles and their correlation with psychological performance. Electroencephalography and Clinical Neurophysiology.

[70] Solisortiz. S, Ramos J, Arce C, Guevara MA, Corsi-Cabrera M (1994). EEG oscillations during menstrual cycle. International Journal of Neuroscience.

[71] Gulsum, A., Emine, F. Y., Gulsen, Y. & Gamze, D. The EEG alpha response is affected by changes in sex hormone levels in two phases of menstrual cycle. *Clinical Neurophysiology* **128**, 10.1016/j.clinph.2017.07.286 (2017)

[72] Sumner RL (2018). Peak visual gamma frequency is modified across the healthy menstrual cycle. Hum Brain Mapp..

[73] Mareike J. Hülsemann, Ewald Naumann & Björn Rasch. Quantification of Phase-Amplitude Coupling in Neuronal Oscillations: Comparison of Phase-Locking Value, Mean Vector Length, Modulation Index, and Generalized-Linear-Modeling-Cross-Frequency-Coupling. *Frontiers in Neuroscience* **13** (2019). 10.3389/fnins.2019.00573 PMC6592221 31275096

[74] Delorme A, Makeig S (2004). EEGLAB: An open source toolbox for analysis of single-trial EEG dynamics including independent component analysis. Journal of Neuroscience Methods.

[75] Thompson T, Steffert T, Ros T, Leach J, Gruzelier J (2008). EEG applications for sport and performance. Methods.

[76] Lachaux J-P, Rodriguez E, Martinerie J, Varela FJ (1999). Measuring phase synchrony in brain signals. Human Brain Mapping.

[77] Varela F, Lachaux J-P, Rodriguez E, Martinerie J (2001). The brainweb: Phase synchronization and large-scale integration. Nature Reviews Neuroscience.

[78] Neubauer AC, Fink A (2009). Intelligence and neural efficiency: Measures of brain activation versus measures of functional connectivity in the brain. Intelligence.

[79] Cohen MX (2015). Effects of time lag and frequency matching on phase-based connectivity. Journal of neuroscience methods.

[80] Stam CJ, Nolte G, Daffertshofer A (2007). Phase lag index: Assessment of functional connectivity from multi channel EEG and MEG with diminished bias from common sources. Human Brain Mapping.

[81] Benjamini Y, Hochberg Y (1995). Controlling the False Discovery Rate: A Practical and Powerful Approach to Multiple Testing. Journal of the Royal Statistical Society B..

[82] Toffanin P, Johnson A, de Jong R, Martens S (2007). Rethinking Neural Efficiency: Effects of Controlling for Strategy Use. Behavioral Neuroscience.

[83] Aydore S, Pantazis D, Leahy RM (2013). A note on the phase locking value and its properties. Neuroimage.

