# Supplementary material for: Resting-state EEG activity predicts frontoparietal network reconfiguration and improved attentional performance
Source: Sci Rep. 2020 Mar 19;10:5064. doi: 10.1038/s41598-020-61866-7 (PMC7081192; doi:10.1038/s41598-020-61866-7)
Supplement: Supplementary file 1 — Supplementary information. [file 41598_2020_61866_MOESM1_ESM.pdf]

Title: Resting-state EEG activity predicts frontoparietal network reconfiguration and improved attentional performance

SUPLEMENTARY INFORMATION

Jacek Rogala, Ewa Kublik, Rafał Krauz, Andrzej Wróbel

Jacek Rogala. Bioimaging Research Center, World Hearing Center, Institute of Physiology and Pathology of Hearing, Mokra 17 street, Kajetany k. Warszawy, 05-830 Nadarzyn, Poland. Email: j.rogala@nencki.edu.pl

Ewa Kublik. Instytut Biologii Doświadczalnej im. Marcelego Nenckiego, 3 Pasteur Street, 02-093, Warsaw, Poland. Email: e.kublik@nencki.gov.pl

Rafał Krauz. Military University of Technology, Physical Education, 3 gen. Sylwestra Kaliskiego street, 00-908, Warsaw, Poland. Email: rafal.krauz@gmail.com

Andrzej Wróbel. Department of Epistemology, Institute of Philosophy, University of Warsaw. 3 Krakowskie Przedmiescie street, 00-927 Warszawa, Poland. Email: andrzej.wrobel@uw.edu.pl

Corresponding author: Jacek Rogala, j.rogala@nencki.edu.pl

S1. Averaged behavioral results of the visual search and shooting tasks and the averaged gB2rest value for all participants

Subject Id	gB2rest	Attention task				Shooting task	
		reaction times (s)		Accuracy (%)		shooting precision scores	
		TEST	RETEST	TEST	RETEST	TEST	RETEST
A9509113	0.79	1.15	1.13	87.50	77.08	271.70	282.30
1656DE8B	0.70	1.31	0.99	89.58	85.42	196.20	254.10
1CA24B1A	0.29	0.88	0.89	83.33	66.67	252.20	311.00
234EF9E7	0.38	0.98	0.71	87.50	87.50	279.40	338.50
275999EA	0.21	1.09	0.86	81.25	89.58	326.10	313.80
2B2E935F	0.24	1.15	1.17	91.67	91.67	268.30	251.10
2D3A067E	0.28	1.19	0.92	56.25	75.00	201.00	235.40
2F24E93F	0.25	1.12	0.79	93.75	87.50	270.00	320.20
2FE454B7	0.61	0.87	0.93	81.25	87.50	223.00	310.40
2FF8AC65	0.72	1.20	1.24	83.33	81.25	252.20	248.40
359EF4B5	0.26	1.13	1.09	83.33	93.75	306.90	323.30
3B3BF9FA	0.53	1.25	1.05	85.42	89.58	242.80	281.00
3E59AC47	0.43	1.21	1.22	93.75	83.33	246.10	249.60
4D11A3CA	0.31	1.29	1.30	79.17	75.00	258.90	293.10
509FE52C	0.35	1.09	0.83	85.42	58.33	262.80	276.60
56F70D3B	0.16	0.87	1.05	64.58	75.00	274.90	333.70
5AC7059E	0.24	0.90	0.89	66.67	68.75	304.60	327.30
5B98A33F	1.02	1.10	1.04	56.25	77.08	255.90	308.80
76E538F7	1.11	1.36	1.32	87.50	89.58	195.40	271.30
7CF103AF	0.78	1.35	1.25	85.42	95.83	233.80	216.40
888B1F99	0.41	1.21	1.19	89.58	93.75	241.40	303.80
8A74A17B	0.40	0.77	1.08	81.25	89.58	267.90	287.10
97C9455E	0.60	1.06	1.04	64.58	79.17	248.30	306.80
9F31060D	0.52	1.02	0.97	89.58	93.75	255.40	289.00
A17E20EF	0.77	1.20	1.20	87.50	87.50	251.90	255.90
A526B66E	0.78	1.29	1.26	75.00	87.50	215.20	297.60
ABED2013	0.39	1.21	1.22	87.50	89.58	216.50	285.60
B14C2F87	0.95	1.21	1.06	77.08	72.92	209.80	283.20
B37A62C7	0.21	1.05	0.98	83.33	91.67	249.30	281.60
C554E0C6	0.40	1.02	0.96	87.50	89.58	267.10	282.30
C9FF2272	0.60	0.96	1.09	77.08	83.33	283.70	309.40
DD17AF31	0.66	1.20	0.93	81.25	85.42	211.90	239.00
E693F5C0	0.65	1.04	1.15	89.58	70.83	284.80	261.70

S2. Correlations between gB2rest and frontoparietal and frontooccipital PLVs in resting-state

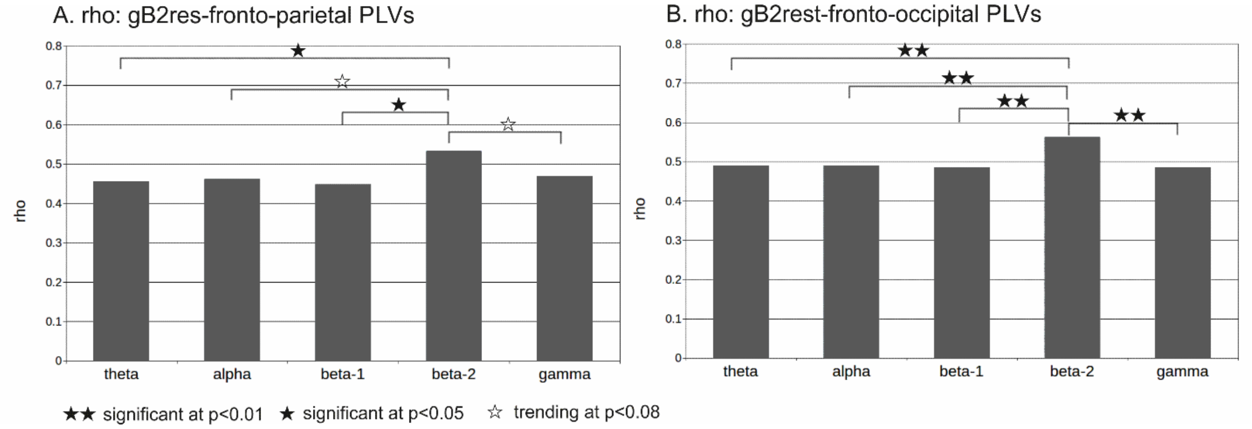

Fig. S2. Averaged correlations between gB2rest and fronto-parietal (A) as well as fronto-occipital (B) PLVs calculated for all investigated bands. Note that for both connections PLVs in the beta-2 band were significantly higher than PLVs in any other EEG band.

In total, frontoparietal PLVs included 101 pairs of signals spanning frontal (F) and centroparietal (CP) as well as frontal (F) and parietal (P) recordings. Frontooccipital PLVs included 39 pairs of signals spanning frontal (F) parietooccipital (PO) and occipital (O) recordings.

For each of the two groups (frontoparietal and frontooccipital) 2-tailed t-test was calculated to compare average PLV differences between investigated bands.

S3. Correlations (Spearman rho) between gB2rest and EEG power during expectation period in visual search

a. theta

test

W1 W2 W3 W4 W5

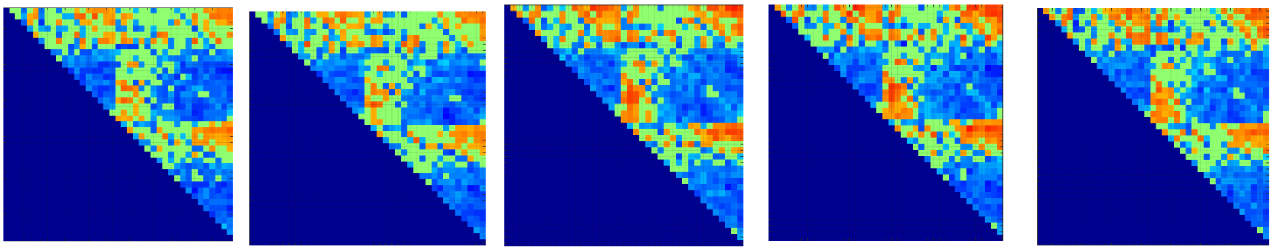

retest

W1 W2 W3 W4 W5

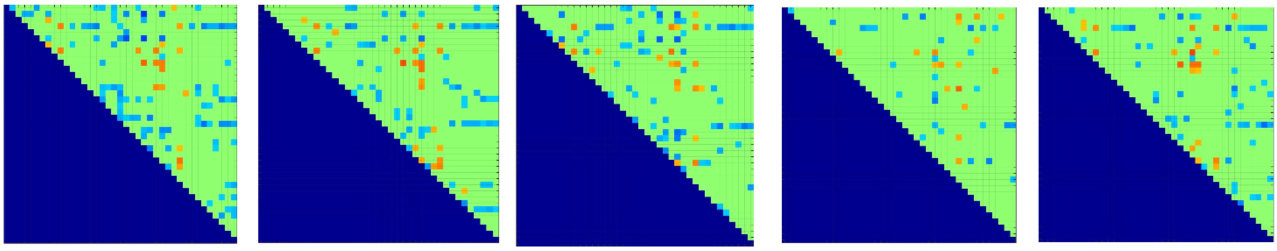

b. alpha

test

W1 W2 W3 W4 W5

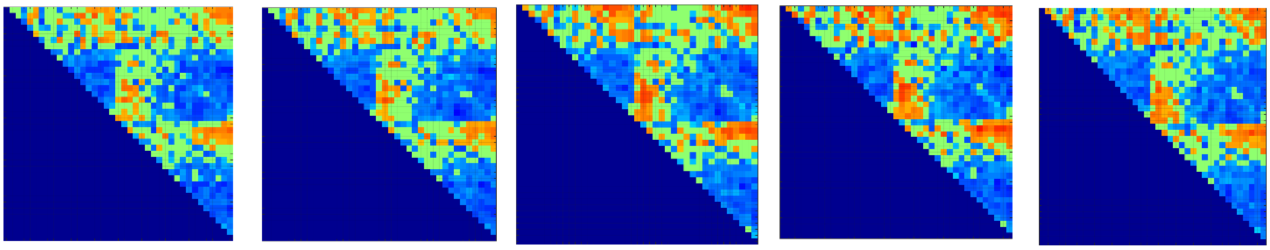

retest

W1 W2 W3 W4 W5

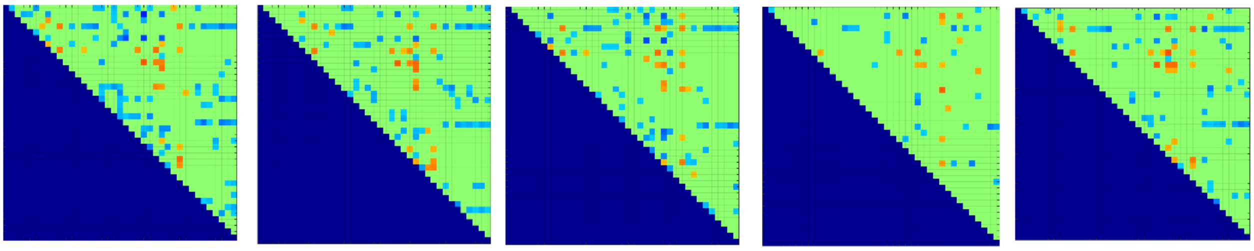

C. beta-1

test

W1 W2 W3 W4 W5

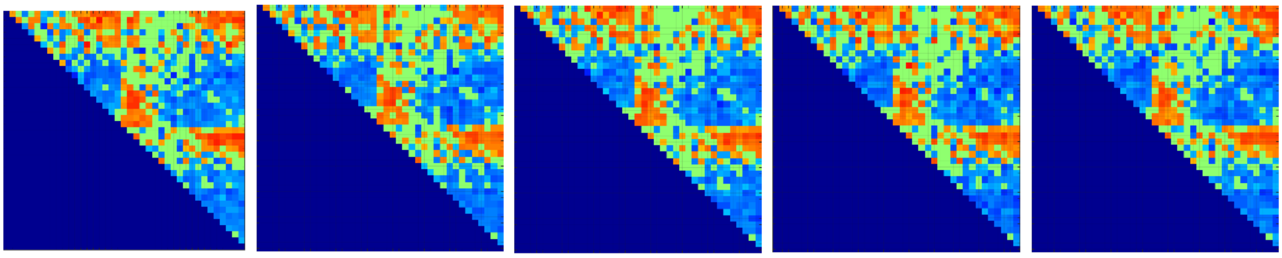

retest

W1 W2 W3 W4 W5

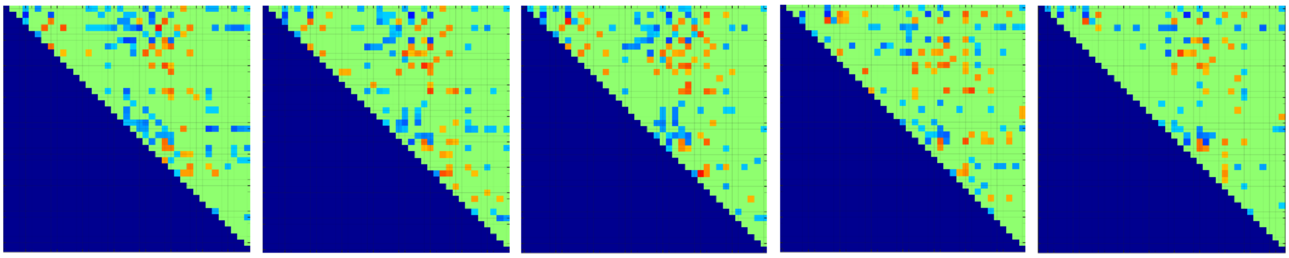

d. beta-2

test

W1 W2 W3 W4 W5

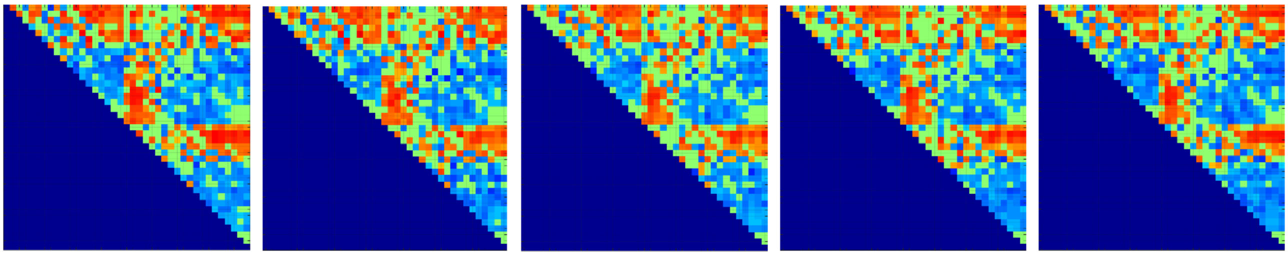

Retest

W1 W2 W3 W4 W5

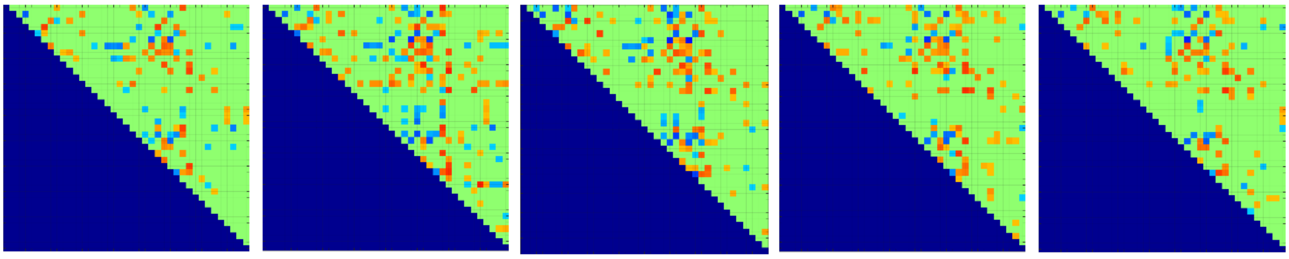

Fig. S3. Correlations between gB2rest and phase locking values in the visual search task. a. theta band, b. alpha band, c. beta-1 band, d. beta-2 band. Each colored square of upper triangular part of matrix denotes either positive (warm colors) or negative (cold colors) or no correlation (green color). W1 …W5 subsequent sliding windows (width 500ms, overlap 200ms). Correlations during test session are significant at p<0.05 FDR corrected. Correlations during retest session are significant at p<0.05 FDR uncorrected. Order of the electrodes: F3, Fz, F4, FC5, FC1, FC2, FC6, C3, C4, CP5, CP1, CP2, CP6, P3, Pz, P4, O1, Oz, O2, F5, F1, F2, F6, FC3, FC4, C5, C1, C2, C6, CP3, CPz, CP4, P5, P1, P2, P6, PO3, POz, PO4.

S4. Correlations (Spearman rho) between gB2rest and EEG power during expectation period in shooting task

a. theta

test

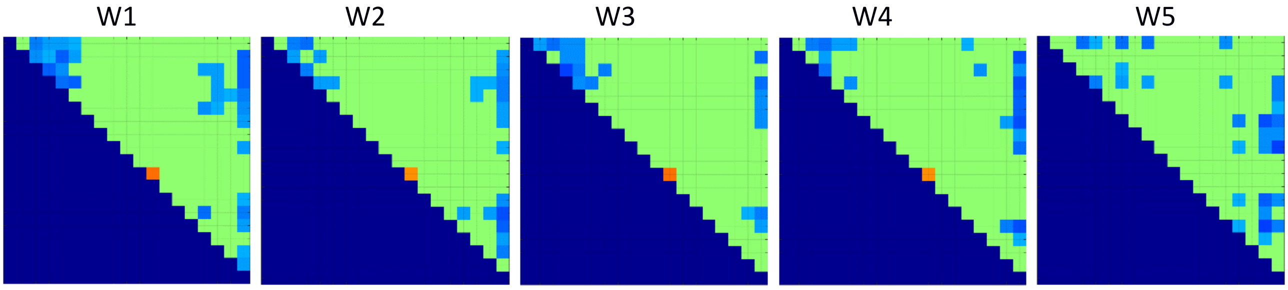

retest

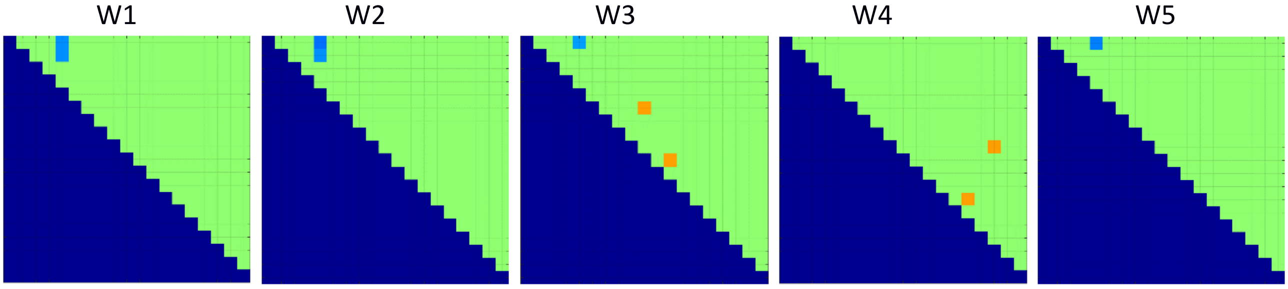

b. alpha

test

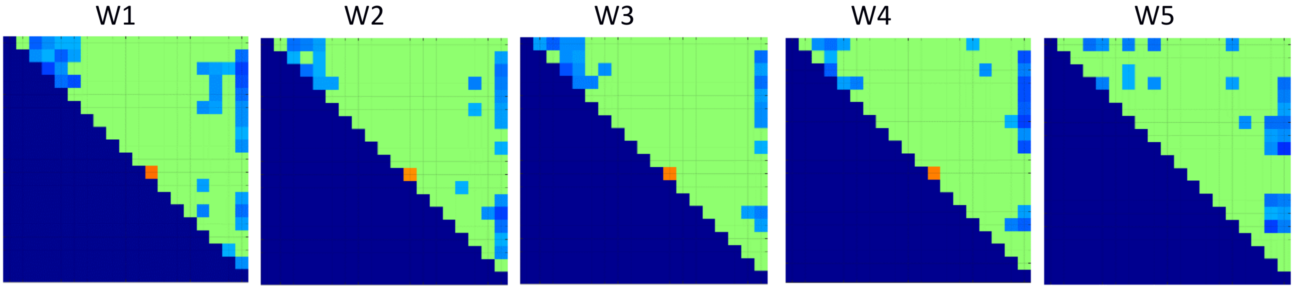

retest

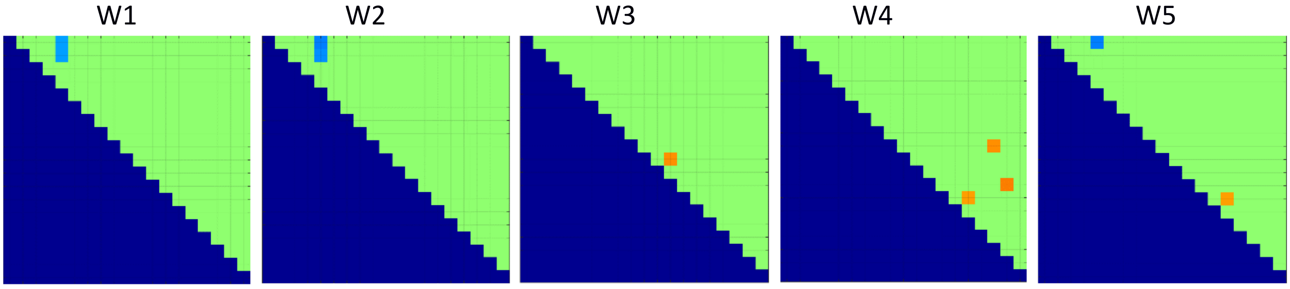

c. beta-1

test

W1 W2 W3 W4 W5

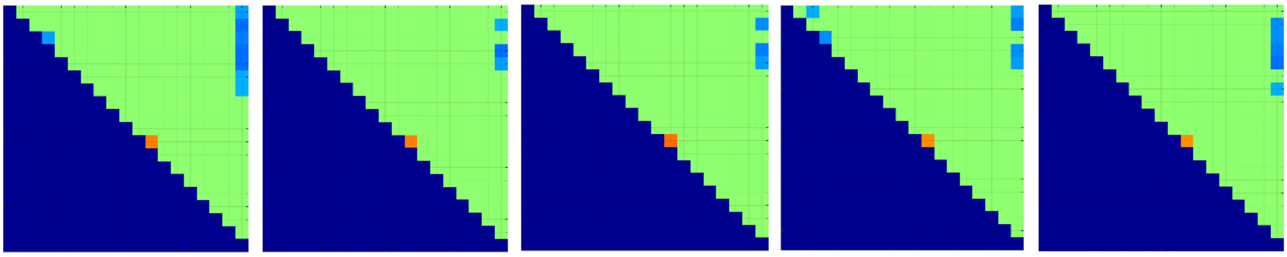

retest

W1 W2 W3 W4 W5

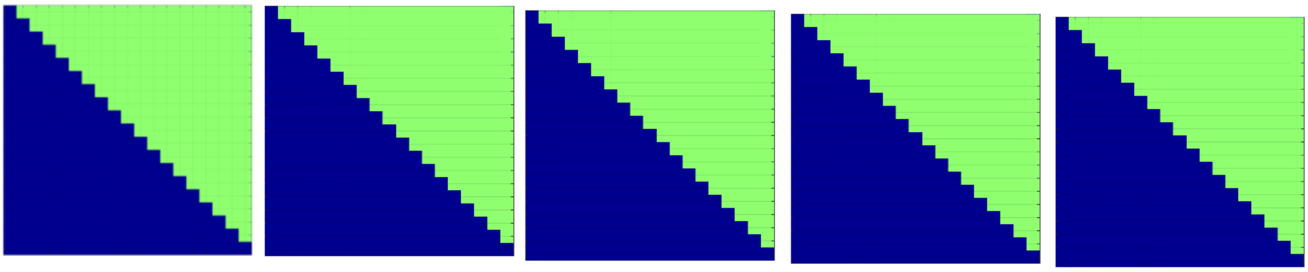

d. beta-2

test

W1 W2 W3 W4 W5

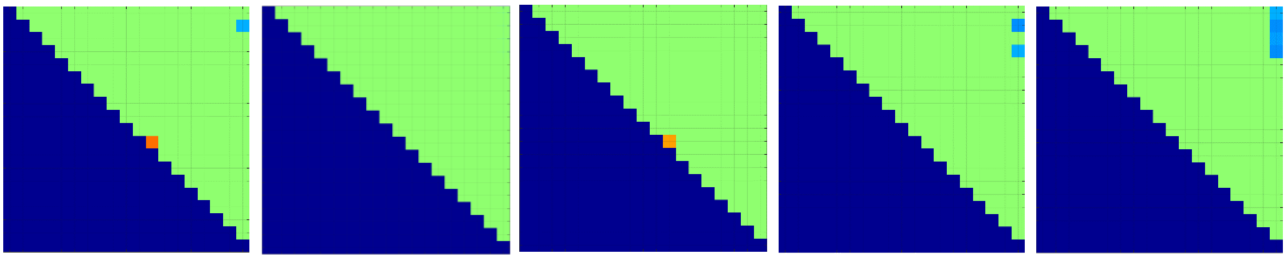

retest

W1 W2 W3 W4 W5

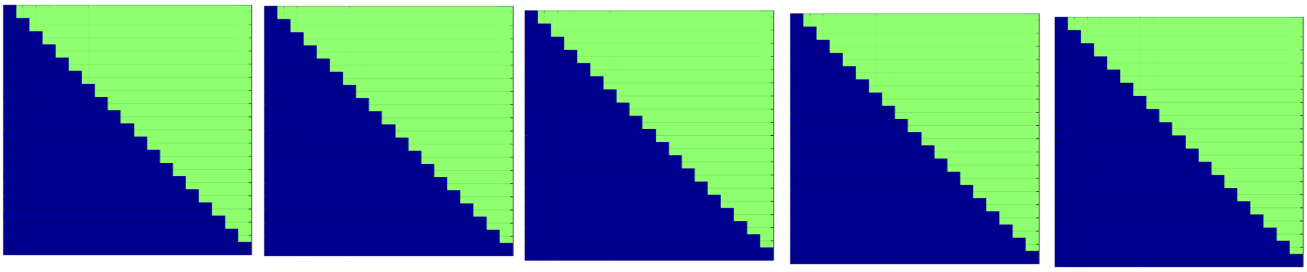

Fig. S4. Correlations between gB2rest and phase locking values in the shooting task. a. theta band, b. alpha band, c. beta-1 band, d. beta-2 band. Each colored square of upper triangular part of matrix denotes either positive (warm colors) or negative (cold colors) or no correlation (green color). W1 …W5 subsequent sliding windows (width 500ms, overlap 200ms). All correlations are significant at $p<0.05$ FDR uncorrected. Order of the electrodes: Fz, F3, FC5, FC1, C3, CP5, CP1, Pz, P3, O1, Oz, O2, P4, CP6, CP2, C4, FC6, FC2, F4.

S5. Number of significant correlations between gB2rest and task related PLV in visual search and shooting task.

a. visual search task

	visual search task: theta band				visual search task: alpha band			
	high gB2rest-low gB2rest		retest-test		high gB2rest-low gB2rest		retest-test	
Window (ms before target onset)	Test	retest	high gB2rest	low gB2rest	test	retest	high gB2rest	low gB2rest
1700-1200	487	22	0	560	489	26	0	559
1400-900	494	0	0	563	496	0	0	564
1100-600	514	9	0	567	513	10	0	567
800-300	507	0	0	550	507	0	0	551
500-0	509	0	0	571	510	0	0	571
**Median**	**507**	**0**	**0**	**563**	**507**	**0**	**0**	**564**
**Wilcoxon test p**	**0,0079**		**0,0079**		**0,0079**		**0,0079**	

	visual search task: beta-1 band				visual search task: beta-2 band			
	high gB2rest-low gB2rest		retest-test		high gB2rest-low gB2rest		retest-test	
Window (ms before target onset)	test	retest	high gB2rest	low gB2rest	test	retest	high gB2rest	low gB2rest
1700-1200	500	53	0	555	509	13	0	551
1400-900	483	37	0	552	493	10	0	554
1100-600	474	43	0	558	473	33	0	549
800-300	480	40	0	553	477	9	0	547
500-0	479	19	0	563	487	9	0	552
**Median**	**480**	**40**	**0**	**555**	**487**	**10**	**0**	**551**
**Wilcoxon test p**	**0,0079**		**0,0079**		**0,0079**		**0,0079**	

b. shooting task

	shooting task: theta band				shooting task: alpha band			
	high gB2rest-low gB2rest		retest-test		high gB2rest-low gB2rest		retest-test	
Window (ms before target onset)	test	retest	high gB2rest	low gB2rest	test	retest	high gB2rest	low gB2rest
1700-1200	37	3	3	15	43	3	5	16
1400-900	35	4	0	10	40	2	0	10
1100-600	22	9	6	11	27	8	6	10
800-300	27	9	18	16	30	9	23	15
500-0	42	3	5	30	44	3	4	24
**Median**	**35**	**4**	**5**	**15**	**40**	**3**	**5**	**15**
**Wilcoxon test p**	**0,0079**		**0,0952**		**0,0022**		**0,0794**	

S6. Similarity between resting-state and task-related connectivity

Pearson r values calculated for PLV maps from resting-state and task-related EEG data

Visual search		
**EEG band**	**test**	**retest**
theta	0.97**	0.88**
alpha	0.97**	0.88**
beta-1	0.97**	0.89**
beta-2	0.98**	0.88**

Shooting		
**EEG band**	**test**	**retest**
theta	0.38**	0.32*
alpha	0.38**	0.32*
beta-1	0.31**	0.29*
beta-2	0.26*	0.26*

** significance at $p<0.01$
* significance at $p<0.05$

S7. Significant PLV differences between gB2rest and task related PLV in visual search task.

a. theta

low gB2rest group in retest minus low gB2rest group in test

W1 W2 W3 W4 W5

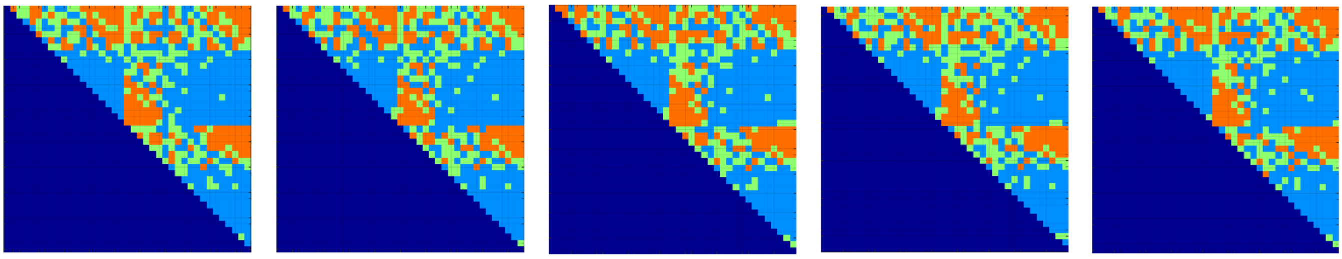

high gB2rest group in retest minus high gB2rest group in test

W1 W2 W3 W4 W5

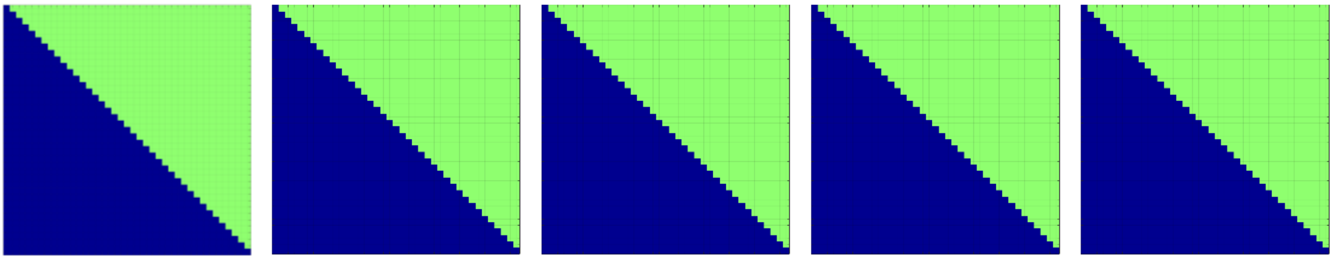

b. alpha

low gB2rest group in retest minus low gB2rest group in test

W1 W2 W3 W4 W5

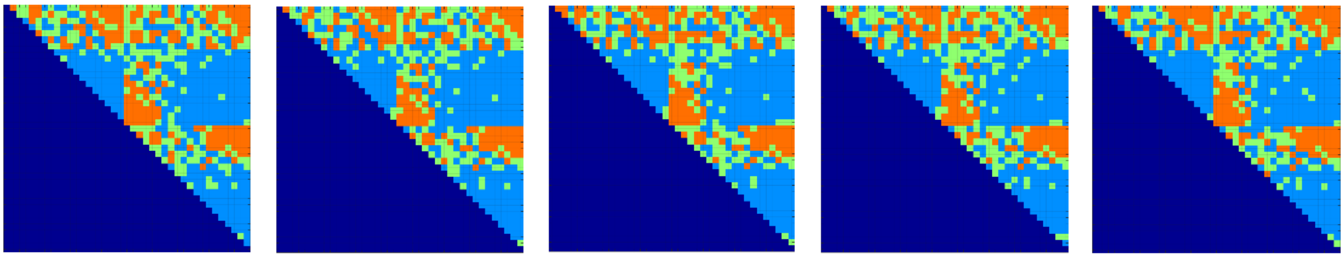

high gB2rest group in retest minus high gB2rest group in test

W1 W2 W3 W4 W5

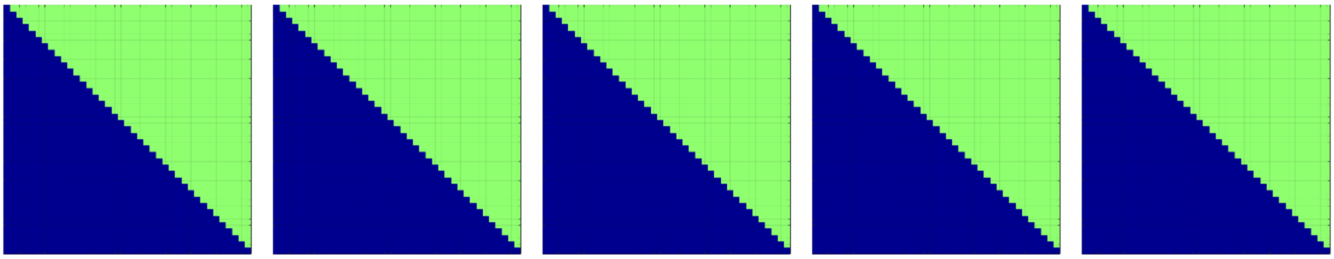

c. beta-1

low gB2rest group in retest minus low gB2rest group in test

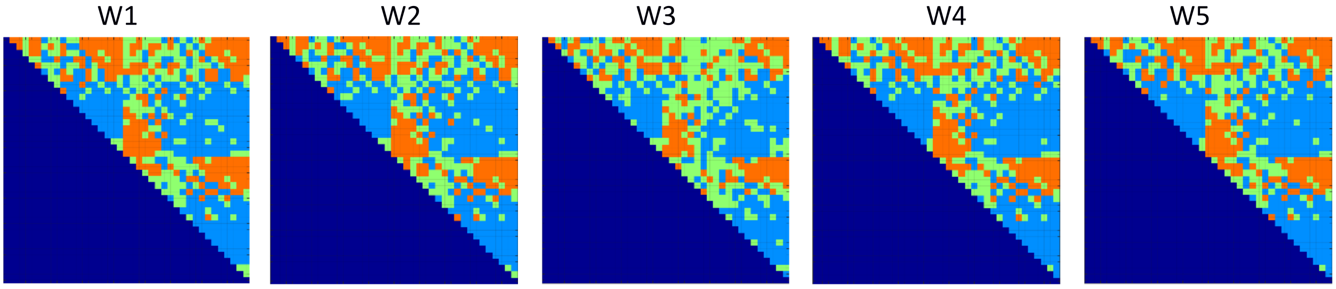

high gB2rest group in retest minus high gB2rest group in test

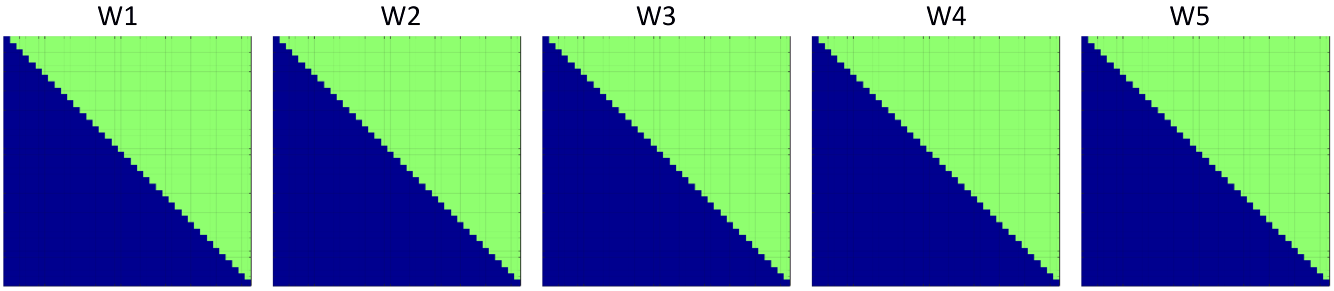

d. beta-2

low gB2rest group in retest minus low gB2rest group in test

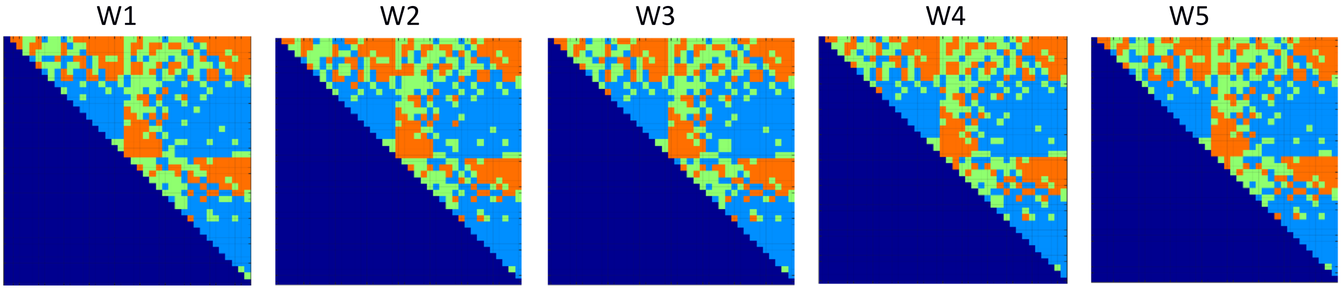

high gB2rest group in retest minus high gB2rest group in test

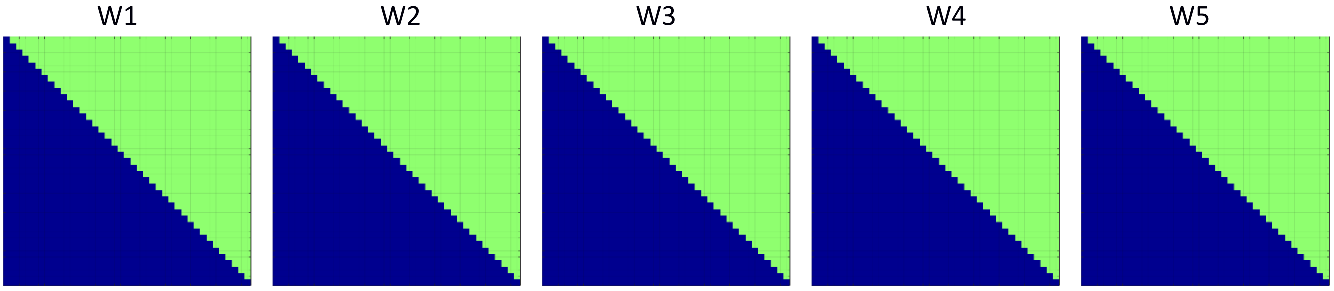

Fig. S7. Group differences between phase locking values in visual search task. a. theta band, b. alpha band, c. beta-1 band, d. beta-2 band. Each colored square of upper triangular part of matrix denotes either positive (red) or negative (blue) or no differences (green color). W1 …W5 subsequent sliding windows (width 500ms, overlap 300ms). All correlations are significant at $p<0.05$ FDR corrected. Order of the electrodes: F3, Fz, F4, FC5, FC1, FC2, FC6, C3, C4, CP5, CP1, CP2, CP6, P3, Pz, P4, O1, Oz, O2, F5, F1, F2, F6, FC3, FC4, C5, C1, C2, C6, CP3, CPz, CP4, P5, P1, P2, P6, PO3, POz, PO4.

S8. Significant PLV differences between gB2rest and task related PLV in shooting task.

a. theta

low gB2rest group in retest minus low gB2rest group in test

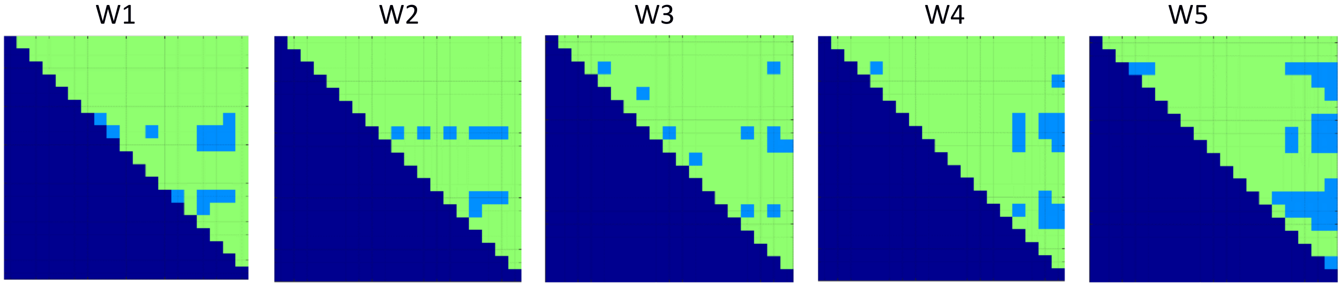

high gB2rest group in retest minus high gB2rest group in test

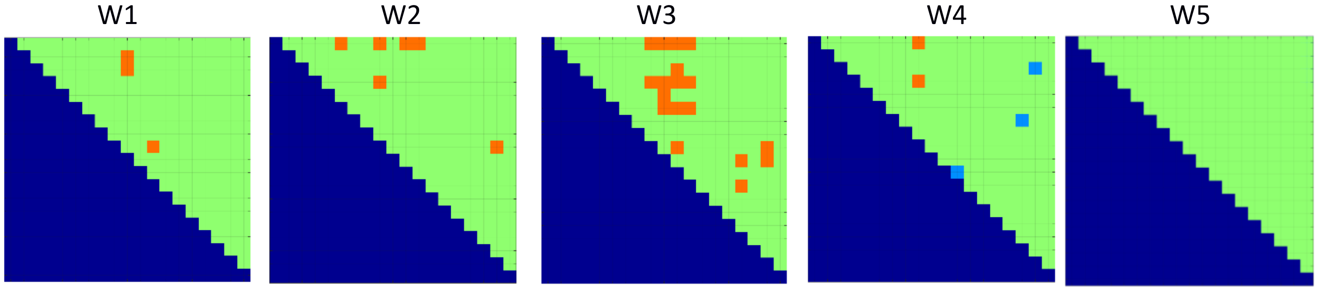

a. alpha

low gB2rest group in retest minus low gB2rest group in test

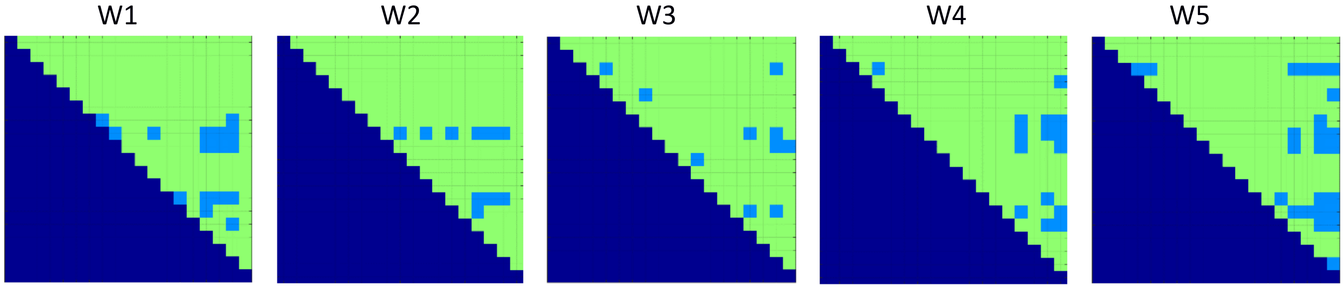

high gB2rest group in retest minus high gB2rest group in test

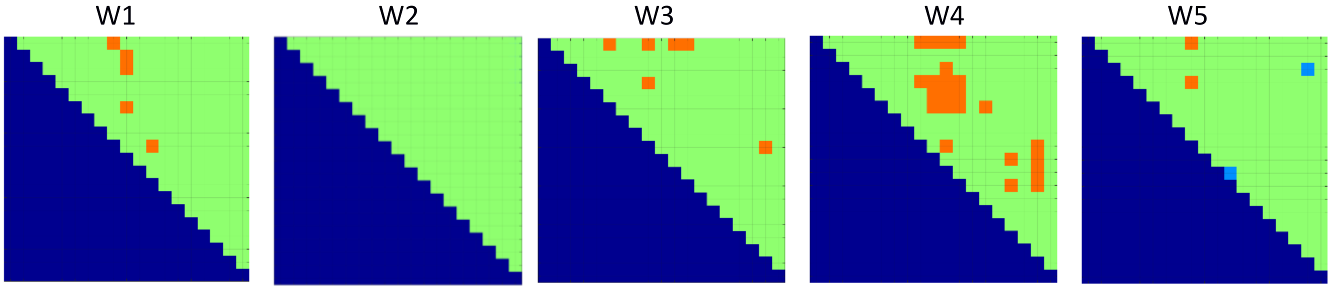

Fig. S8. Group differences between phase locking values in shooting task. a. theta band, b. alpha band. Each colored square of upper triangular part of matrix denotes either positive (red) or negative (blue) or no differences (green color). W1 …W5 subsequent sliding windows (width 500ms, overlap 200ms). All correlations are significant at $p<0.05$ uncorrected. Order of the electrodes: Fz, F3, FC5, FC1, C3, CP5, CP1, Pz, P3, O1, Oz, O2, P4, CP6, CP2, C4, FC6, FC2, F4.

S9. Correlations (Spearman rho) between gB2rest and EEG power during expectation period in visual search and shooting task

Visual search									Shooting*							
theta		alpha		beta-1		beta-2		el.	Theta		alpha		beta-1		beta-2	
Test	retest	test	retest	test	retest	test	retest		test	retest	test	retest	test	retest	test	retest
0.25	0.28	0.50	0.21	0.65	0.70	0.77	0.60	**F3**	0.08	0.14	-0.09	0.41	0.13	0.30	0.02	0.26
0.46	0.33	0.54	0.11	0.71	0.80	0.82	0.61	**Fz**	-0.23	-0.03	-0.21	0.25	-0.04	0.24	-0.16	0.24
0.17	0.03	0.62	-0.01	0.70	0.71	0.77	0.69	**F4**	-0.02	0.12	0.11	0.36	0.16	0.28	0.05	0.46
-0.05	0.10	0.36	0.42	0.42	0.69	0.32	0.67	**FC5**	-0.01	0.00	-0.19	0.55	-0.07	0.28	-0.04	0.16
0.52	0.28	0.65	0.29	0.73	0.78	0.78	0.69	**FC1**	-0.14	-0.17	-0.24	0.23	-0.02	0.24	-0.13	0.28
0.48	0.20	0.68	0.18	0.70	0.70	0.81	0.75	**FC2**	0.17	0.42	0.21	0.03	0.01	0.26	-0.10	0.32
-0.21	0.09	0.14	0.13	0.56	0.77	0.37	0.77	**FC6**	0.21	0.14	0.25	-0.10	-0.06	0.19	-0.03	0.23
0.23	0.24	0.52	0.41	0.58	0.81	0.41	0.73	**C3**	-0.23	0.15	-0.13	0.27	0.19	0.37	0.03	0.19
-0.07	0.12	0.34	0.42	0.53	0.53	0.46	0.59	**C4**	0.10	0.13	0.18	-0.01	0.00	0.11	-0.02	0.16
-0.25	0.19	0.18	0.39	0.41	0.72	0.28	0.69	**CP5**	0.12	-0.17	-0.19	0.34	0.09	0.32	0.08	0.36
-0.09	-0.06	0.52	0.31	0.57	0.65	0.35	0.74	**CP1**	0.26	-0.03	-0.01	0.45	0.08	0.28	0.11	0.22
-0.07	0.17	0.38	0.43	0.49	0.50	0.37	0.64	**CP2**	0.23	0.05	0.20	0.08	-0.06	0.02	-0.02	0.28
-0.21	0.25	0.06	0.28	0.27	0.65	0.00	0.55	**CP6**	0.18	0.27	0.17	0.29	0.03	0.17	0.02	0.18
-0.26	0.15	0.19	0.19	0.51	0.58	0.22	0.63	**P3**	-0.16	-0.13	0.10	0.16	0.13	0.17	0.08	0.38
-0.19	0.15	0.22	0.17	0.41	0.43	0.16	0.65	**Pz**	0.13	-0.01	0.07	0.49	0.10	0.31	0.05	0.29
-0.28	0.29	0.11	0.23	0.34	0.55	0.08	0.52	**P4**	0.03	0.28	0.13	0.42	0.01	0.25	0.03	0.26
-0.32	-0.05	-0.06	0.02	0.22	0.36	0.11	0.12	**O1**	-0.15	0.15	0.14	0.14	-0.01	0.22	0.04	-0.06
-0.34	0.00	-0.13	0.10	0.11	0.49	0.01	0.11	**Oz**	0.17	0.00	0.11	0.18	0.10	0.19	-0.04	0.22
-0.33	0.14	-0.09	0.16	0.08	0.52	-0.09	0.31	**O2**	0.14	0.13	0.08	0.40	0.00	0.22	0.07	0.24
-0.04	0.06	0.42	0.29	0.55	0.73	0.56	0.70	**F5**								
0.42	0.37	0.54	0.14	0.76	0.78	0.81	0.63	**F1**								
0.39	0.23	0.67	0.07	0.73	0.80	0.83	0.69	**F2**								
-0.08	0.15	0.19	0.02	0.47	0.67	0.59	0.76	**F6**								
0.39	0.24	0.60	0.36	0.72	0.73	0.61	0.75	**FC3**								
0.11	0.11	0.43	0.20	0.76	0.70	0.71	0.72	**FC4**								
-0.16	0.14	0.21	0.44	0.44	0.76	0.27	0.74	**C5**								
0.40	0.08	0.63	0.44	0.76	0.78	0.50	0.76	**C1**								
0.16	0.11	0.53	0.35	0.77	0.62	0.70	0.70	**C2**								
-0.21	0.12	0.04	0.30	0.44	0.72	0.24	0.68	**C6**								
-0.18	0.25	0.36	0.29	0.51	0.67	0.37	0.56	**CP3**								
-0.09	-0.05	0.41	0.24	0.55	0.58	0.40	0.69	**CPz**								
-0.08	0.30	0.30	0.36	0.40	0.52	0.28	0.67	**CP4**								
-0.36	0.25	0.11	0.15	0.46	0.51	0.19	0.58	**P5**								
-0.22	0.14	0.22	0.22	0.43	0.55	0.21	0.70	**P1**								
-0.22	0.29	0.24	0.16	0.41	0.44	0.12	0.53	**P2**								
-0.27	0.36	0.06	0.35	0.22	0.63	-0.15	0.49	**P6**								
-0.29	0.17	0.05	0.07	0.34	0.49	0.12	0.44	**PO3**								
-0.32	0.14	0.08	0.10	0.31	0.56	0.04	0.51	**POz**								
-0.36	0.26	-0.05	0.18	0.21	0.65	-0.04	0.46	**PO4**								

Significant correlation coefficients are in red (p<0.05. FDR corrected).

S10. test-retest differences in power of four EEG bands during expectation period in visual search task

El.	theta				alpha				beta1				beta2			
	HIGH		LOW		HIGH		LOW		HIGH		LOW		HIGH		LOW	
	T	R	T	R	T	R	T	R	T	R	T	R	T	R	T	R
**F3**	2,500	2,372	1,693	2,014	2,200	2,435	2,012	2,088	1,599	1,735	1,057	1,129	0,766	0,886	0,484	0,472
**Fz**	2,881	3,107	1,909	3,140	2,616	3,084	1,950	2,762	1,724	1,928	0,896	1,285	0,713	0,796	0,346	0,502
**F4**	2,344	2,392	1,760	2,071	2,015	2,399	1,914	2,254	1,533	1,720	1,041	1,131	0,751	0,812	0,482	0,493
**FC5**	1,859	1,613	2,052	1,019	1,806	1,877	2,571	1,606	1,130	1,127	1,064	0,687	0,545	0,535	0,508	0,291
**FC1**	1,887	2,131	1,031	1,900	1,692	2,138	1,317	2,026	1,119	1,387	0,641	0,954	0,488	0,596	0,262	0,381
**FC2**	1,803	1,995	0,983	1,906	1,776	2,099	1,625	2,289	1,129	1,324	0,673	0,938	0,488	0,584	0,275	0,390
**FC6**	1,917	1,452	1,982	1,059	1,849	1,865	2,320	1,701	1,180	1,082	1,136	0,763	0,537	0,493	0,514	0,329
**C3**	1,511	1,474	1,499	1,093	2,493	2,629	3,192	2,563	1,488	1,511	1,195	1,064	0,563	0,546	0,428	0,338
**C4**	1,810	1,603	1,647	1,195	3,180	3,268	3,253	2,880	1,686	1,552	1,261	1,070	0,608	0,561	0,484	0,378
**CP5**	2,366	1,955	2,652	1,153	2,892	3,182	4,249	2,404	1,552	1,372	1,587	0,923	0,582	0,513	0,615	0,325
**CP1**	1,725	1,420	1,656	1,181	2,668	3,129	3,855	3,092	1,218	1,326	1,292	1,005	0,412	0,407	0,391	0,277
**CP2**	1,819	1,477	1,702	1,315	3,171	3,681	4,209	3,497	1,266	1,272	1,306	1,028	0,418	0,415	0,419	0,291
**CP6**	2,044	1,653	2,810	1,180	2,922	2,704	4,121	2,586	1,530	1,325	1,699	0,922	0,555	0,471	0,635	0,329
**P3**	2,360	1,773	2,913	1,578	3,631	3,898	5,208	3,416	1,876	1,665	2,024	1,304	0,596	0,519	0,689	0,392
**Pz**	2,598	2,026	2,663	1,891	4,718	6,096	6,324	4,567	1,698	1,697	1,935	1,382	0,523	0,488	0,593	0,364
**P4**	2,313	1,857	3,226	1,743	4,075	4,609	5,633	3,724	1,856	1,737	2,224	1,334	0,600	0,535	0,766	0,391
**O1**	4,367	2,813	4,989	2,782	4,755	3,500	5,635	3,778	3,942	2,253	3,245	2,003	2,106	1,030	1,707	1,013
**Oz**	3,248	2,578	4,689	2,408	3,001	2,920	5,478	3,159	2,096	1,940	2,964	1,621	1,131	0,937	1,571	0,798
**O2**	3,239	2,549	4,867	2,419	3,156	3,052	6,119	3,435	2,046	1,823	3,560	1,773	1,024	0,801	2,185	0,931
**F5**	2,456	1,887	2,084	1,511	2,176	2,125	2,415	1,947	1,543	1,470	1,185	0,978	0,863	0,919	0,593	0,435
**F1**	2,640	2,666	1,721	2,734	2,353	2,663	1,784	2,425	1,627	1,917	0,875	1,217	0,693	0,806	0,347	0,479
**F2**	2,543	2,806	1,786	2,660	2,249	2,761	1,945	2,570	1,624	1,824	0,951	1,221	0,714	0,765	0,392	0,495
**F6**	2,322	1,953	2,243	1,566	1,873	2,156	2,285	1,982	1,453	1,461	1,229	1,035	0,821	0,813	0,619	0,485
**FC3**	1,680	1,653	1,322	1,309	1,799	1,961	1,994	1,760	1,174	1,278	0,884	0,836	0,534	0,578	0,381	0,328
**FC4**	1,704	1,659	1,243	1,330	2,115	2,198	1,976	2,150	1,267	1,274	0,883	0,925	0,560	0,548	0,379	0,381
**C5**	1,892	1,703	2,227	1,011	2,356	2,396	3,464	1,948	1,342	1,194	1,251	0,769	0,527	0,460	0,520	0,292
**C1**	1,526	1,555	1,093	1,285	1,930	2,276	2,337	2,532	1,047	1,243	0,849	0,928	0,385	0,457	0,280	0,293
**C2**	1,638	1,546	1,078	1,430	2,322	2,523	2,569	2,991	1,095	1,151	0,845	0,959	0,404	0,439	0,286	0,325
**C6**	1,972	1,623	2,301	1,060	2,480	2,406	3,081	2,035	1,414	1,253	1,383	0,824	0,547	0,480	0,570	0,323
**CP3**	1,775	1,366	2,006	1,044	2,691	2,719	4,068	2,665	1,538	1,379	1,499	1,040	0,559	0,473	0,499	0,313
**CPz**	2,123	1,654	1,715	1,403	3,243	3,914	4,118	3,460	1,177	1,277	1,252	0,987	0,386	0,398	0,383	0,280
**CP4**	1,853	1,478	2,190	1,182	3,184	3,227	4,167	2,892	1,567	1,440	1,564	1,006	0,540	0,492	0,548	0,323
**P5**	3,088	2,318	3,493	1,914	4,160	4,196	5,183	3,724	2,197	1,807	2,232	1,427	0,716	0,592	0,863	0,470
**P1**	2,253	1,792	2,651	1,600	3,800	4,566	5,562	3,886	1,641	1,606	1,903	1,278	0,511	0,487	0,601	0,357
**P2**	2,305	1,819	2,729	1,692	4,568	5,527	6,025	4,173	1,662	1,618	1,965	1,301	0,511	0,481	0,624	0,347
**P6**	2,672	2,126	4,042	1,990	3,917	4,055	6,228	4,371	2,027	1,776	2,634	1,531	0,668	0,561	0,998	0,506
**PO3**	3,057	2,432	4,250	2,499	3,798	4,085	6,239	4,439	2,148	1,930	2,678	1,723	0,850	0,648	1,046	0,584
**POz**	2,808	2,181	3,947	2,166	3,461	4,172	6,280	3,801	1,758	1,690	2,477	1,412	0,643	0,559	0,939	0,472
**PO4**	2,995	2,372	4,270	2,347	3,677	4,077	6,094	3,866	1,928	1,820	2,779	1,602	0,718	0,609	1,163	0,573

Significant differences between test and retest are marked with colors (blue for decrease and red increase). T – test, R – retest.

S11. Behavioral differences between groups with high and low global beta-2 resting state power (gB2rest)

To verify whether detected correlations between gB2rest and behavioral performance yielded significant differences we divided all subjects into two equally numbered groups (HIGH and LOW, each containing 16 participants with highest and lowest beta-2 global values, respectively) and compared their behavioral results in TEST and RETEST.

The ANOVA analysis of the reaction times in the attention task showed significant effect of the group ($F(1,30) = 5.5104$ $p=0.0257$). Subsequent Tukey post-hoc test showed trending shorter reaction times of the LOW beta-2 group in both experiments in TEST (diff: 0.1 s; $p=0.0562$) and RETEST (diff: 0.11 s; $p<0.0508$). Significant effect of interaction between groups and sessions ($F(1,30) = 5.3273$, $p=0.0281$) indicated also better performance improvement of the LOW group. HIGH group shortened average RT by 0.0581 s and LOW group by 0.0629 respectively.

The analysis of the shooting scores showed significant effect of the group ($F(1,30) = 7.3565$, $p=0.011$). Subsequent Tukey post-hoc test confirmed significantly higher average number of precision points in the LOW than HIGH beta-2 group in TEST (diff: 26 points; $p=0.0209$) and RETEST (diff: 22 points; $p=0.0394$). Significant effect of interaction between groups and sessions ($F(1,30) = 6.7990$, $p=0.0141$) indicated larger improvement of performance of the HIGH group. The HIGH group increased their precision results by 36 points while LOW group by 32 points. Both differences between sessions were significant ($p<0.01$, all subjects participated in the shooting lessons).

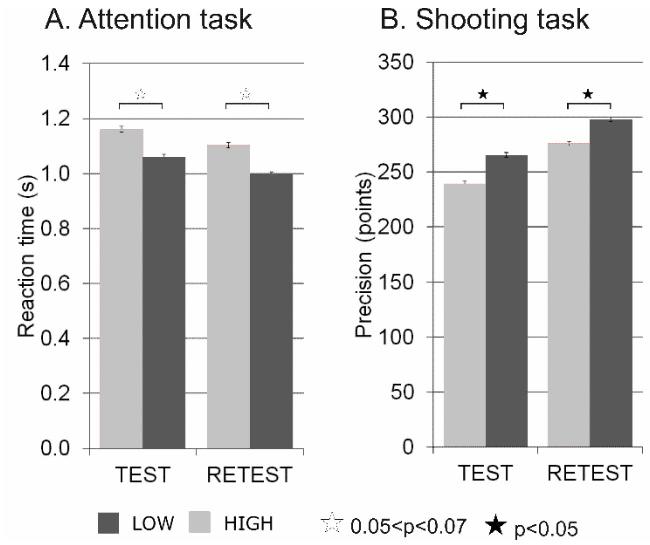

Fig. S11. Reaction times (A) and precision scores (B) measured for HIGH and LOW group in TEST and RETEST. Filled stars indicate differences at p<0.05 and empty stars at p<0.01.

S12. Exclusion of the possible effects of impedance and volume conduction

While high and low groups were defined on the basis of beta-2 powers it came out that their resting state power spectra differed also in the whole range from 2 to 45 Hz (p<0.01, FDR corrected for all electrodes). This result could be attributed to differences in impedance of the electrodes for particular subjects resulting from the differences of conductivity or electrodes impedance. It appeared, however, that the average impendences of the electrodes measured for high and low groups did not differ (two-sample t-test; p=0.22). Additionally, we calculated average impendence value from all electrodes of each subject and selected equally numbered (16 subjects each) subgroups with high and low impedance values. Then we calculated correlations and ANOVA analyses for reaction times and corresponding ERP/FFT/PLV data comparing high and low impendence groups. None of these analyses yielded significant results precluding influence of impendence or conductivity on analyzed parameters.

S13. Electrode locations and regions of interest used for defining behaviorally relevant EEG bands

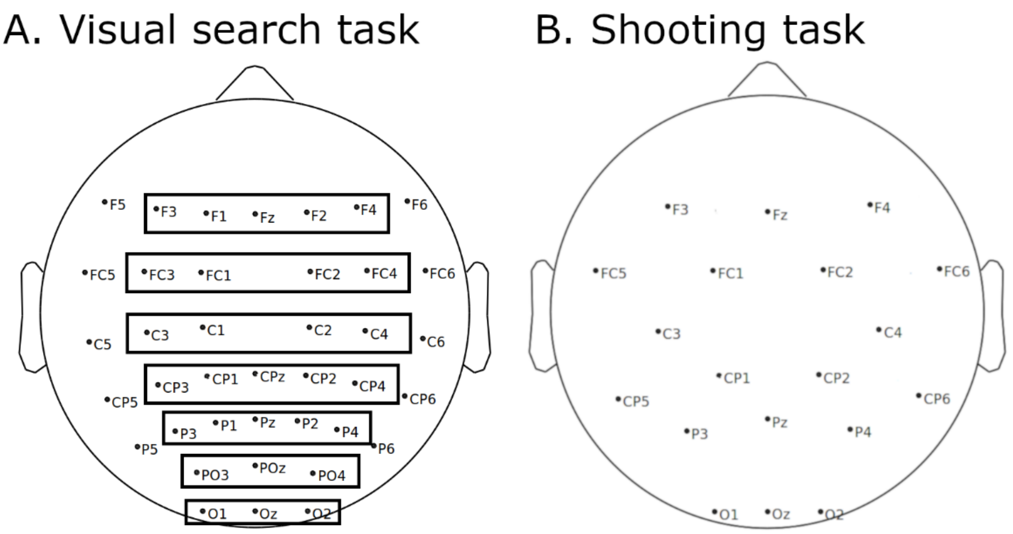

Fig. S13. A. Locations of electrodes and regions of interest used in visual search task data analyses. Each set of outlined electrodes formed separate region of interest (ROI) and their grouped signals were averaged for analyses. The ROIs were labeled (anterior to posterior) as follows: frontal (F). fronto–central (FC). central (C). centro–parietal (CP). parietal (P). parieto–occipital (PO) and occipital (O). (Adopted from Toffanin et al. 2007). B. Locations of electrodes used in shooting task data analyses.

S14. Connectivity analyses with use of phase lag index (PLI)

Comparison of the test-retest connectivity differences with use of the phase lag index showed higher number of significant differences between retest and test in the low gb2rest group than in the high one (except for the alpha band) concordant with results obtained with PLV method. Due to lesser sensitivity of the phase lag index than the phase locking value the number of the pairs of electrodes showing significant (uncorrected) retest to retest differences is much smaller than the results obtained by PLV method.

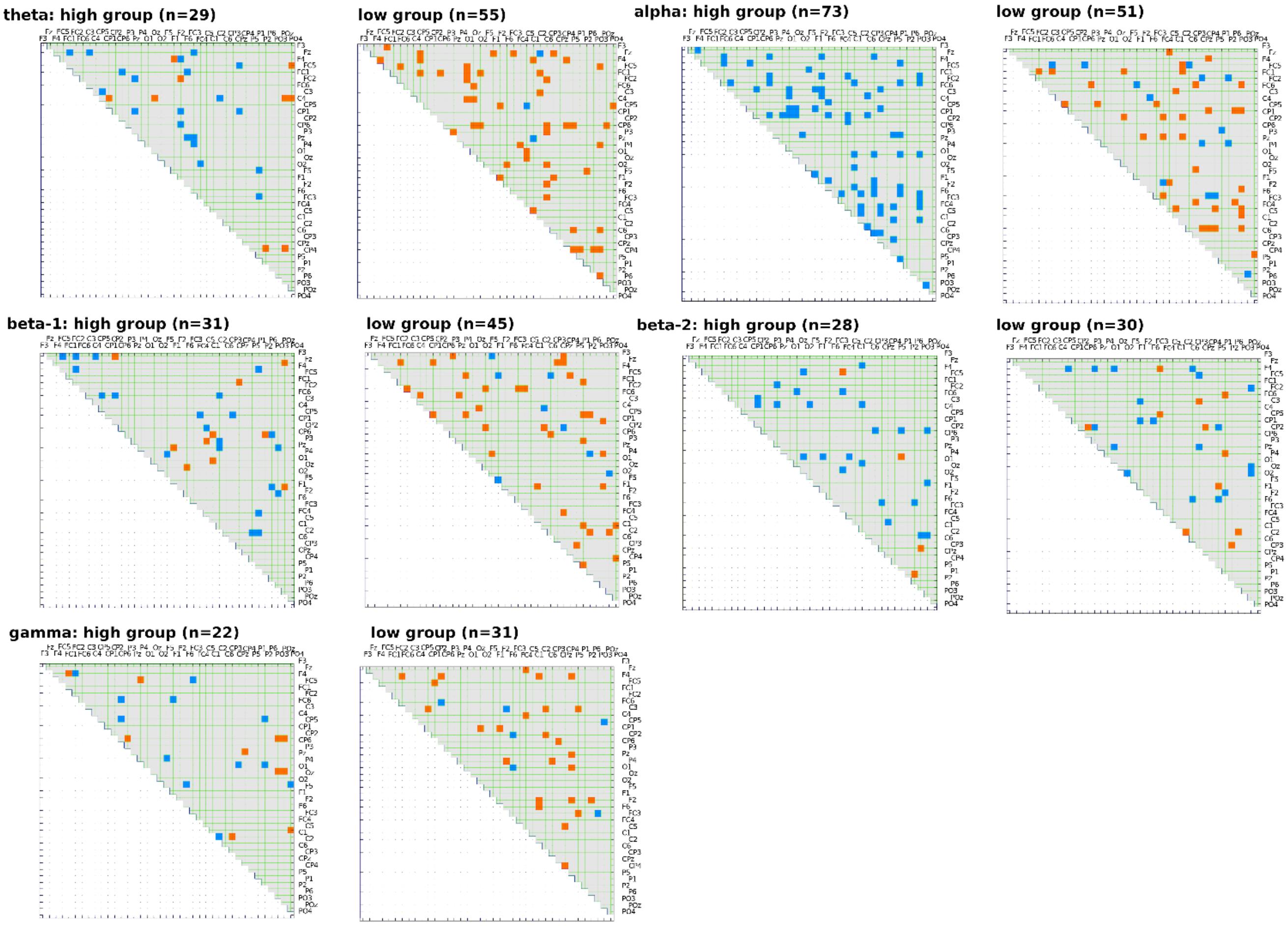

Fig. S14. Significant (p<0.05 uncorrected) differences between retest and test phase lag indices (PLI) in the high and low groups in five EEG bands (theta, alpha, beta-1, beta-2 and gamma). Numbers in brackets show number of electrode pairs which PLI changed from test to retest.
